# Supplementary material for: Comparative analyses of Netherton syndrome patients and Spink5 conditional knock-out mice uncover disease-relevant pathways
Source: Commun Biol. 2024 Feb 5;7:152. doi: 10.1038/s42003-024-05780-y (PMC10844249; doi:10.1038/s42003-024-05780-y)
Supplement: Supplementary file 2 — Supplementary Information [file 42003_2024_5780_MOESM2_ESM.pdf]

Supplementary Information

Comparative analyses of Netherton syndrome patients and *Spink5* conditional knock-out mice uncover disease-relevant pathways

Evgeniya Petrova[1*], Jesús María López-Gay[2,3], Matthias Fahrner[4], Florent Leturcq[1], Jean-Pierre de Villartay[5], Claire Barbieux[1], Patrick Gonschorek[6], Lam C Tsoi[7,8,9], Johann E Gudjonsson[7], Oliver Schilling[4] and Alain Hovnanian[1, 10,11*]

[1]INSERM UMR 1163, Laboratory of Genetic Skin Diseases, Imagine Institute and University of Paris, Paris, France.
[2]Institut Curie, PSL Research University, CNRS UMR 3215, INSERM U934, F-75248 Paris Cedex 05, France.
[3]Sorbonne University, UPMC University Paris 06, CNRS, CNRS UMR 3215, INSERM U934, F-75005 Paris, France.
[4]Institute for Surgical Pathology, Medical Center, Faculty of Medicine, University of Freiburg, Germany; German Cancer Consortium (DKTK) and Cancer Research Center (DKFZ), Freiburg, Germany
[5]Imagine Institute, Laboratory "Genome Dynamics in the Immune System", INSERM UMR 11635, Paris, France.
[6]Institute of Chemical Sciences and Engineering, School of Basic Sciences, Ecole Polytechnique Fédérale de Lausanne (EPFL), Lausanne CH-1015, Switzerland.
[7]Department of Dermatology, University of Michigan Medical School, Ann Arbor, MI, USA.
[8]Department of Computational Medicine & Bioinformatics, University of Michigan Medical School, Ann Arbor, MI, USA.
[9]Department of Biostatistics, School of Public Health, University of Michigan, Ann Arbor, MI, USA.
[10]Department of Genomic Medicine of rare diseases, Necker Hospital for Sick Children, Assistance Publique des Hôpitaux de Paris (AP-HP), Paris, France.
[11]University of Paris Cité, Paris, France

*Correspondence: Evgeniya Petrova and Alain Hovnanian, INSERM UMR 1163, Laboratory of Genetic Skin Diseases, Imagine Institute, 24 Boulevard du Montparnasse, Paris 75015, France.
E-mail: evgeniya.petrova@inserm.fr ; alain.hovnanian@inserm.fr

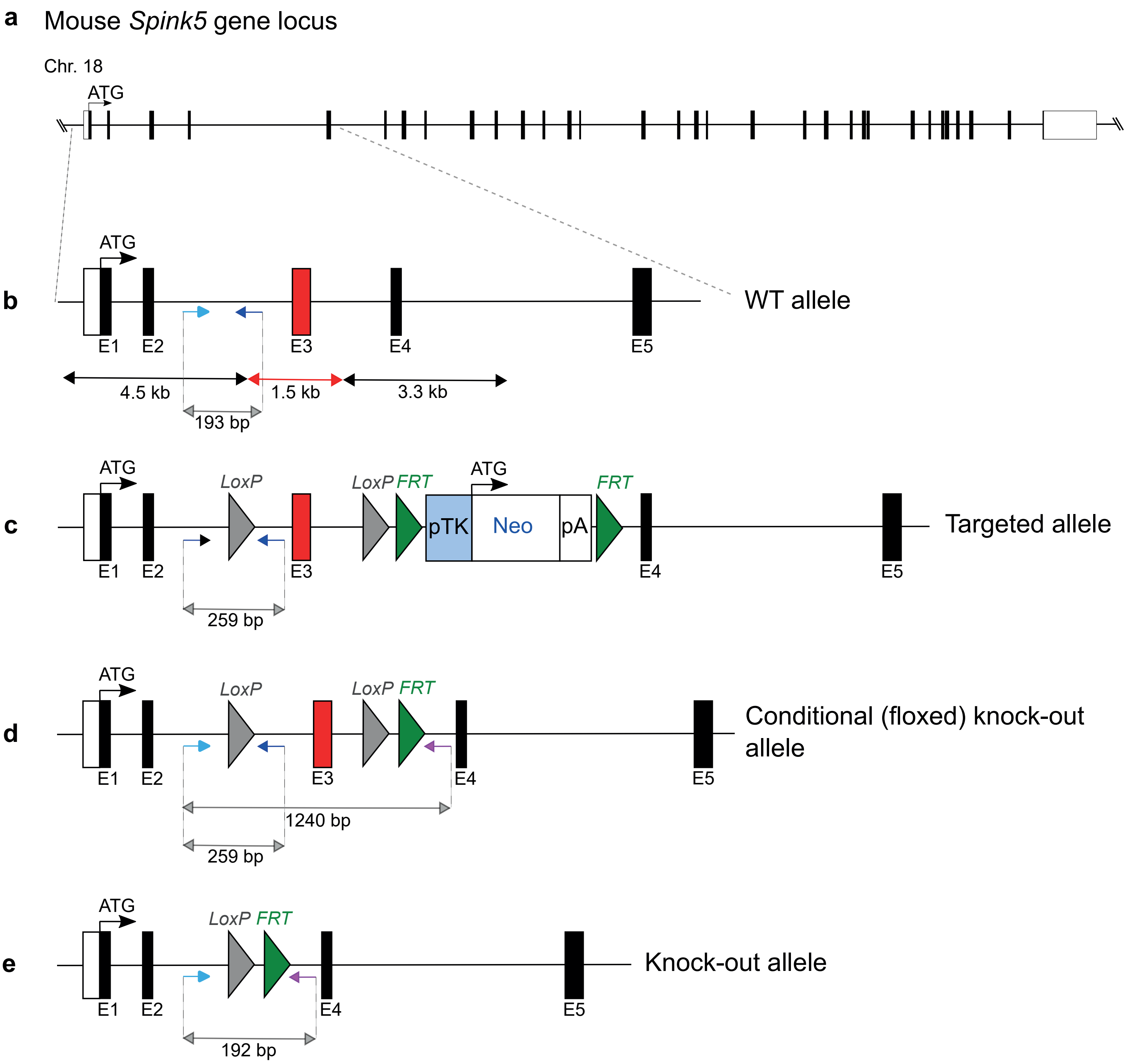

Supplementary Fig. 1: Targeting strategy for conditional inactivation of *Spink5* gene.
a Scheme of mouse *Spink5* gene locus with a close-up (**b**) on the first five exons of *Spink5* and the gene targeting strategy employed. The *Spink5* WT allele (**b**), the targeted allele containing the FRT-flanked (green arrowheads) neomycin resistance gene (**c**), the conditional allele resulting from FLP-mediated excision of the Neo cassette (**d**) and the knock-out allele resulting from Cre-mediated excision of exon 3 are depicted (**e**). Exons (black/white boxes) are labelled (E1, 2, 3, 4,...). Exon 3 marked in red (**b-d**) is excised upon CreERT2-mediated recombination of LoxP sites (grey) (**e**). After tamoxifen-induced Cre-mediated excision of the *loxP*-flanked exon 3, splicing from exon 2 to exon 4 is predicted to induce a frame shift and a premature stop codon. To increase the level of *Spink5* ablation by tamoxifen-inducible CreRT2-mediated floxed allele excision, *KRT14-CreERT2*$^{(Tg/0)}$/*Spink5*$^{fl/fl}$ mice were crossed to the previously described *Spink5*$^{+/-}$ mice, which carry a constitutive *Spink5* knock-out allele, thus resulting in *KRT14-CreERT2*$^{(Tg/0)}$/*Spink5*$^{fl/-}$ mice. Red and black double-headed arrows (**b**) indicate the location and length of the targeted genomic region (exon 3) and the 5' and 3' homology arms used for homologous recombination-mediated insertion of the gene-targeting cassette, respectively. Cyan, blue and magenta arrows indicate the location and combination of the three primers used for PCR-mediated detection of each allele in genomic DNA samples. The length of the corresponding PCR products is indicated by double-headed grey arrows.

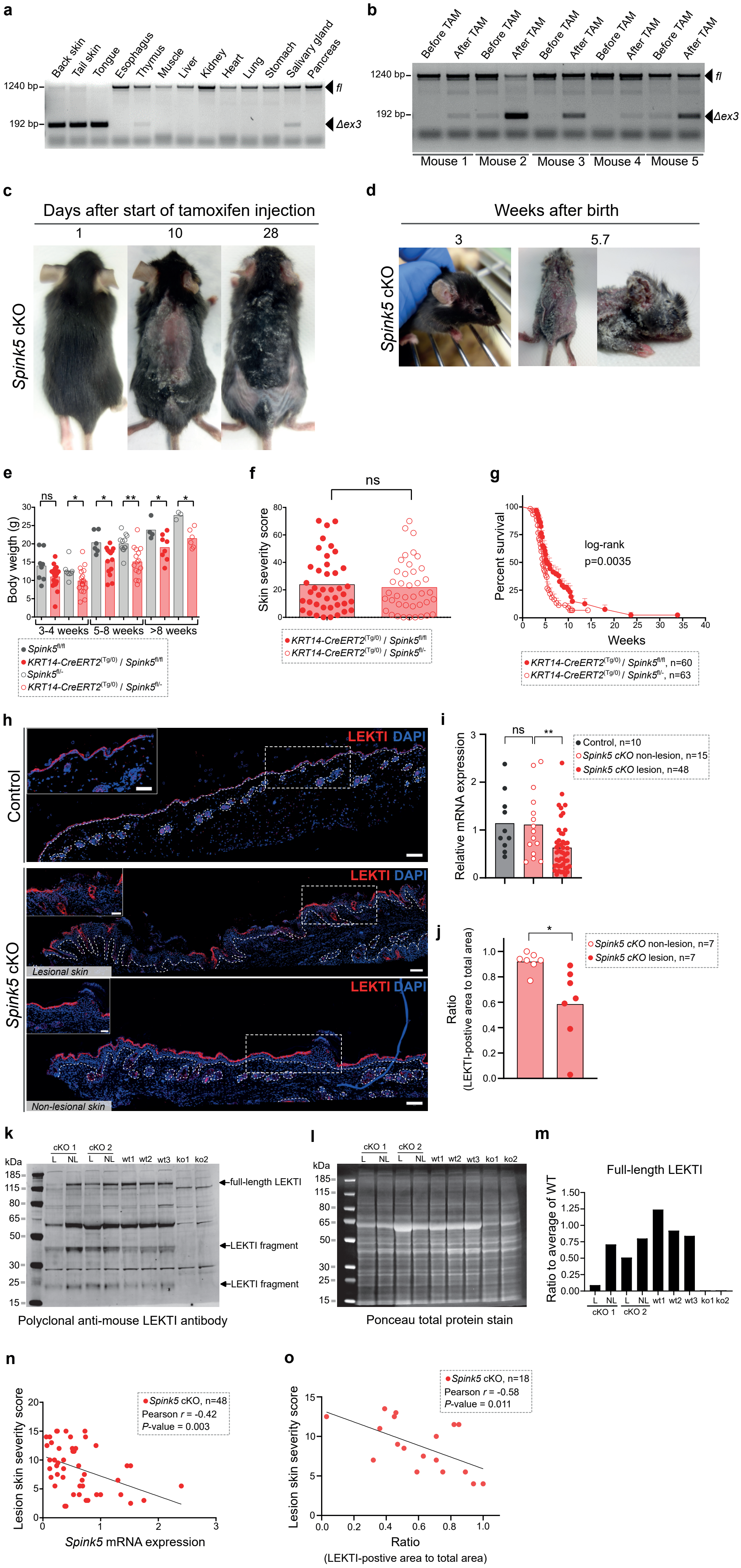
a
Back skin
Tail skin
Tongue
Esophagus
Thymus
Muscle
Liver
Kidney
Heart
Lung
Stomach
Salivary gland
Pancreas
1240 bp
192 bp
fl
Δex3
b
Before TAM
After TAM
Mouse 1
Mouse 2
Mouse 3
Mouse 4
Mouse 5
c
Days after start of tamoxifen injection
1
10
28
Spink5 cKO
d
Weeks after birth
3
5.7
Spink5 cKO
e
Body weigth (g)
3-4 weeks
5-8 weeks
>8 weeks
ns
Spink5fl/fl
KRT14-CreERT2(Tg/0) / Spink5fl/fl
Spink5fl/-
KRT14-CreERT2(Tg/0) / Spink5fl/-
f
Skin severity score
ns
KRT14-CreERT2(Tg/0) / Spink5fl/fl
KRT14-CreERT2(Tg/0) / Spink5fl/-
g
Percent survival
Weeks
log-rank
p=0.0035
KRT14-CreERT2(Tg/0) / Spink5fl/fl, n=60
KRT14-CreERT2(Tg/0) / Spink5fl/-, n=63
h
Control
Spink5 cKO
LEKTI DAPI
Lesional skin
Non-lesional skin
i
Relative mRNA expression
ns
**
Control, n=10
Spink5 cKO non-lesion, n=15
Spink5 cKO lesion, n=48
j
Ratio
(LEKTI-postive area to total area)
Spink5 cKO non-lesion, n=7
Spink5 cKO lesion, n=7
k
kDa
cKO 1
cKO 2
L
NL
wt1
wt2
wt3
ko1
ko2
full-length LEKTI
LEKTI fragment
LEKTI fragment
Polyclonal anti-mouse LEKTI antibody
l
Ponceau total protein stain
m
Full-length LEKTI
Ratio to average of WT
n
Lesion skin severity score
Spink5 mRNA expression
Spink5 cKO, n=48
Pearson r = -0.42
P-value = 0.003
o
Lesion skin severity score
Ratio
(LEKTI-postive area to total area)
Spink5 cKO, n=18
Pearson r = -0.58
P-value = 0.011

Supplementary Fig. 2: Conditional deletion of *Spink5* results in NS-like skin phenotype.

a Image of agarose gel electrophoresis of amplified PCR products from genomic DNA for detection of CreERT2-mediated *Spink5* exon 3 excision in different tissue samples collected from an individual *Spink5* conditional knock-out mouse (these analyses are for a mouse different from the one shown in Fig.1c, demonstrating inter-individual variability in the level of floxed allele excision). **b** Image of agarose gel electrophoresis of amplified PCR products from genomic DNA for detection of CreERT2-mediated *Spink5* exon 3 excision in tail skin of *Spink5* cKO mice before and after tamoxifen (TAM) administration. **c** Images of back skin of a *Spink5* cKO mouse showing the appearance of skin lesions over time following tamoxifen administration. **d** Images of a *Spink5* cKO mouse with spontaneous, tamoxifen-independent *Spink5* floxed allele excision showing the evolution of skin phenotype with age. **e** Bar plot of body weight across different age groups of control mice (grey) and *Spink5* cKO mice (red) carrying homozygous *Spink5* conditional alleles (*KRT14-CreERT2*$^{(Tg/0)}$/*Spink5*$^{fl/fl}$, filled circles) and heterozygous *Spink5* conditional and constitutive knock-out alleles (*KRT14-CreERT2*$^{(Tg/0)}$/*Spink5*$^{fl/-}$, open circles). **f** Bar plot of whole body skin severity scores measured at time of sacrifice in *Spink5* cKO mice (*KRT14-CreERT2*$^{(Tg/0)}$/*Spink5*$^{fl/fl}$, filled circles and *KRT14-CreERT2*$^{(Tg/0)}$/*Spink5*$^{fl/-}$, open circles). **g** Kaplan-Meier survival curve analysis of *Spink5* cKO mice (*KRT14-CreERT2*$^{(Tg/0)}$/*Spink5*$^{fl/fl}$, filled circles, and *KRT14-CreERT2*$^{(Tg/0)}$/*Spink5*$^{fl/-}$, open circles). **h** Tile scan image of LEKTI immunofluorescence staining of an entire back skin section from a control mouse (upper panel) and a seven-week-old *Spink5* cKO littermate mouse with spontaneous CreERT2 activation (lesional skin and non-lesional (mild) skin, middle and lower panels). The dermal-epidermal junction is outlined with a white dashed line. Scale bars: 100 µm. A white dashed-line rectangle outlines the zoomed area in the inset. Scale bars: 50 µm. **i** Bar plot of *Spink5* mRNA relative expression ratios measured in back skin samples from control mice and in lesional and non-lesional back skin samples from *Spink5* cKO mice. **j** Bar plot of LEKTI protein expression levels measured in non-lesional and lesional back skin of *Spink5* cKO mice. LEKTI protein expression levels are represented as the ratio of LEKTI-positive epidermis area to total epidermis area measured in images of LEKTI immunofluorescence stainings. **k** SDS-PAGE/Western blot analysis of LEKTI expression in lesional (L) and non-lesional (NL) back skin of two different *Spink5* cKO mice (cKO 1 and cKO 2), three different WT control mice (wt1, wt2 and wt3) and two different *Spink5*$^{-/-}$ mice (ko1 and ko2). Skin extracts from WT mice and *Spink5*$^{-/-}$ mice were used as positive and negative controls, respectively. Black arrows indicate bands corresponding to full-length LEKTI (120 kDa) and to possible LEKTI proteolytic fragments (40 kDa and 24 kDa). **l** Image of the membrane shown in (**k**) stained with Ponceau. **m** Bar plot of full-length LEKTI band intensities from the membrane in (**k**) normalized to total protein levels (Ponceau stain) and expressed as ratio to the average of the full-length LEKTI bands in WT control mice. **n** Scatter plot of the correlation between *Spink5* mRNA expression level in skin of *Spink5* cKO mice (fold change relative to control mice) and lesional skin severity score of *Spink5* cKO mice. Pearson correlation coefficient *r* and statistical significance (*P*-value) are indicated on the plot. **o** Scatter plot of the correlation between LEKTI protein levels and severity score of lesional back skin. LEKTI protein expression levels in lesional back skin samples are represented as the ratio of LEKTI-positive epidermis area to total epidermis area measured in images of LEKTI immunofluorescence stainings. Pearson correlation coefficient *r* and statistical significance (*P*-value) are indicated on the plot.

Data in (**a**) is representative of three independent experiments. Data in (**c-d**) is representative of at least 10 independent experiments. Data in (**e-f**) and (**i-j**) are means (bars) and scatter plots, where dots correspond to individual mice ($n \geq 3$ per group). Data in (**k-m**) is representative of experiments done with 4 *Spink5* cKO mice (lesional and non-lesional back skin samples), 4 control WT mice and 2 *Spink5*$^{-/-}$ mice. Statistical significance was determined using two-tailed non-parametric Wilcoxon matched-pairs signed rank test (in **e**, **f** and **j**), log-rank test (**g**) or Mann-Whitney test (**i**): *$p<0.05$, **$p<0.01$. Control mice are *Spink5*$^{fl/fl}$ and/or *Spink5*$^{fl/-}$; *Spink5* cKO mice are *KRT14-CreERT2*$^{(Tg/0)}$/*Spink5*$^{fl/fl}$ and/or *KRT14-CreERT2*$^{(Tg/0)}$/*Spink5*$^{fl/-}$.

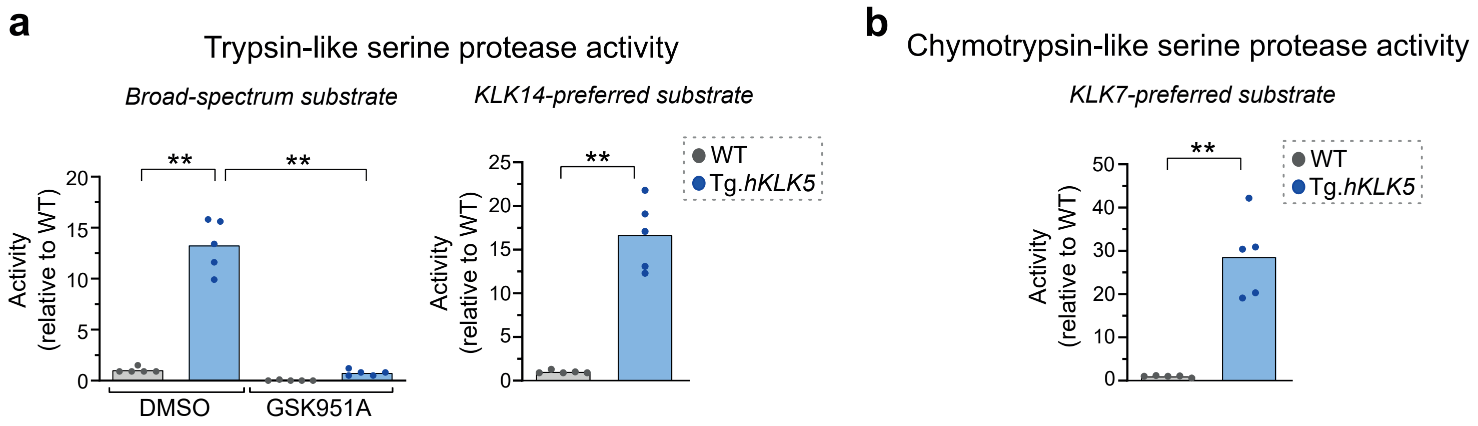

Supplementary Fig. 3: Measurement of protease activity in back skin of Tg.*hKLK5* mice.

a Measurements of trypsin-like serine protease activity in protein extracts from back skin of control (grey) and Tg.*hKLK5* (blue) mice using the broad-spectrum fluorogenic substrate for trypsin-like serine proteases Boc-VPR-amc (left panel) and the KLK14-preferred fluorogenic peptide substrate Ac-WAVR-amc (right panel). **b** Measurements of chymotrypsin-like serine protease activity in protein extracts from back skin of control mice (grey) and Tg.*hKLK5* mice (blue) using the KLK7-preferred fluorogenic peptide substrate KHLY-amc. In (**a**) and (**b**), activity is expressed as a ratio of the fluorescence intensity value measured in each skin sample to the mean of the fluorescence intensity values measured in skin extracts from control mice. The addition of the KLK5-specific inhibitor GSK951A serves as a control to estimate the percent of trypsin-like protease activity due to KLK5 activation. DMSO was added as a negative control for protease activity inhibition. The graphs in (**a**) and (**b**) show means (bars) and scatter plots, where data points correspond to individual mice (n=5). Each dot represents the mean of all measurements for an individual mouse. Statistical significance was determined using a two-tailed non-parametric Mann-Whitney test: **p<0.01.

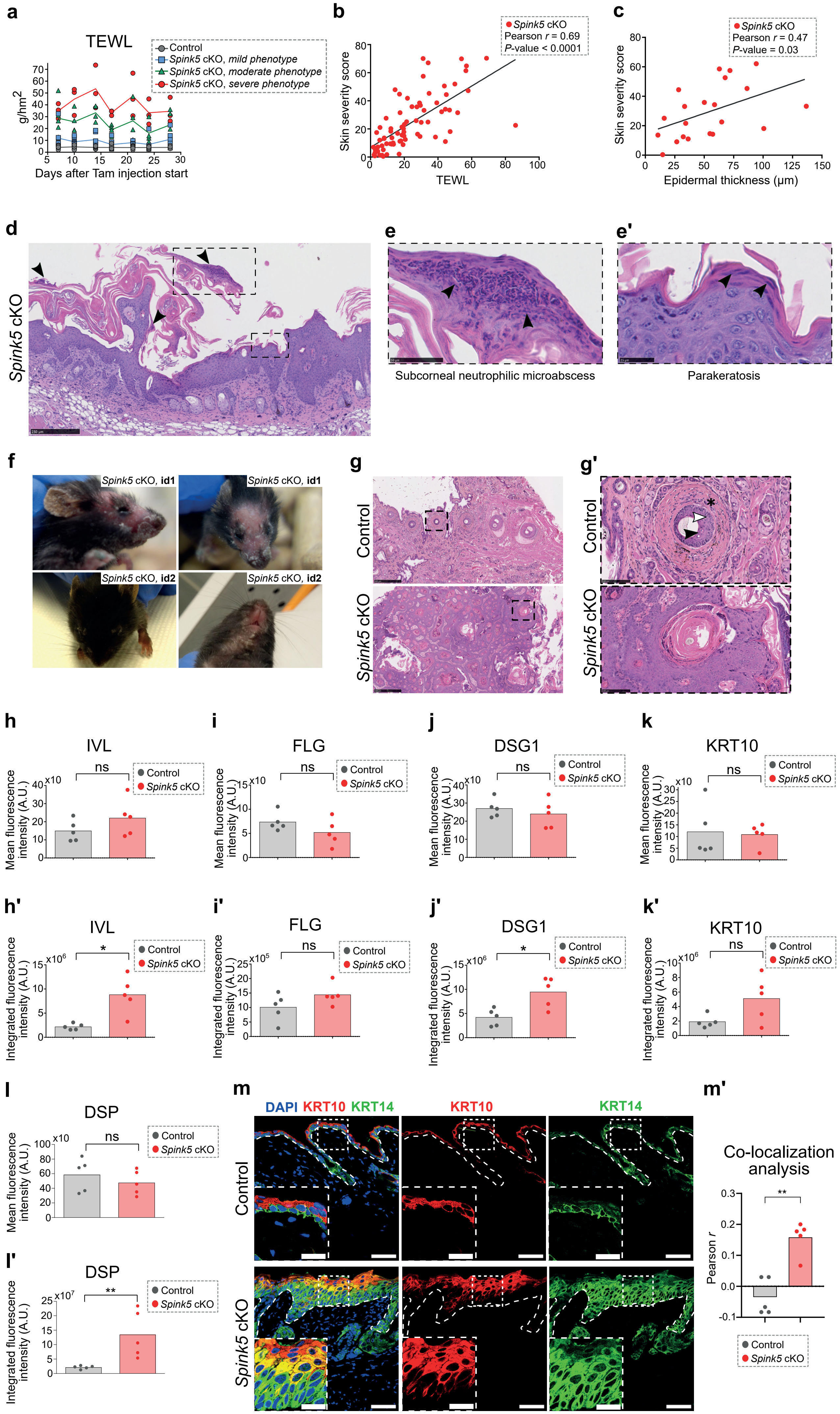

a
TEWL
Control
Spink5 cKO, mild phenotype
Spink5 cKO, moderate phenotype
Spink5 cKO, severe phenotype
g/hm2
Days after Tam injection start
b
Spink5 cKO
Pearson r = 0.69
P-value < 0.0001
Skin severity score
TEWL
c
Spink5 cKO
Pearson r = 0.47
P-value = 0.03
Skin severity score
Epidermal thickness (µm)
d
Spink5 cKO
e
Subcorneal neutrophilic microabscess
e'
Parakeratosis
f
Spink5 cKO, id1
Spink5 cKO, id1
Spink5 cKO, id2
Spink5 cKO, id2
g
Control
Spink5 cKO
g'
Control
Spink5 cKO
h
IVL
i
FLG
j
DSG1
k
KRT10
Mean fluorescence intensity (A.U.)
ns
Control
Spink5 cKO
h'
IVL
i'
FLG
j'
DSG1
k'
KRT10
Integrated fluorescence intensity (A.U.)
*
ns
l
DSP
Mean fluorescence intensity (A.U.)
l'
DSP
Integrated fluorescence intensity (A.U.)
**
m
DAPI KRT10 KRT14
KRT10
KRT14
Control
Spink5 cKO
m'
Co-localization analysis
Pearson r
**
Control
Spink5 cKO

Supplementary Fig. 4: Characterization of skin barrier and epidermal differentiation defects in *Spink5* conditional knock-out mice.

a Transepidermal water loss (TEWL) values measured over three weeks following tamoxifen administration on shaved back skin of control mice (grey) and *Spink5* cKO mice with mild (blue squares), moderate (green triangles) and severe (red circles) phenotypes. **b** Scatter plot of the correlation between whole body skin severity score and TEWL measured on back skin of *Spink5* cKO mice (n=76). **c** Scatter plot of the correlation between epidermal thickness and whole-body skin severity score measured in *Spink5* cKO mice (n=21). In (**b**) and (**c**), regression line, Pearson correlation coefficient *r* and *P*-value are indicated on each plot. **d** Hematoxylin and eosin staining of lesional back skin from the *Spink5* cKO mouse shown in Fig. 2d. Filled black arrowheads point to a subcorneal neutrophilic microabscess. Black dashed-line rectangles denote magnified areas shown in panels (**e-e'**). Scale bar: 250 µm. **e-e'** Magnified images of regions outlined with a black dashed-line rectangles in panel (**d**). Filled black arrowheads point to subcorneal neutrophilic microabscess (**e**) and parakeratosis (nucleated keratinocytes in the stratum corneum) (**e'**). Scale bars: 50 µm, 25 µm. **f** Images of muzzle skin of a *Spink5* cKO mouse with severe skin lesions (upper panels, id1) and a *Spink5* cKO mouse with mild skin lesions (lower panels, id2). Vibrissae hairs (whiskers) were absent in some *Spink5* cKO mice with spontaneous floxed allele excision. **g-g'** Hematoxylin and eosin staining of muzzle skin cross sections from control mice (upper panel) and *Spink5* cKO (lower panel) littermate mice. The whisker hair follicles in *Spink5* cKO mice are larger and disorganized with no distinction between inner root sheath and outer root sheath. The *Spink5* cKO hair follicles are surrounded by hyperplastic epidermis and are filled with keratinized debris and neutrophilic microabscesses. Black dashed-line rectangles denote magnified areas displayed in (**g'**). Filled arrowhead points to inner root sheath. Open arrowhead points to outer root sheath (**g'**). The location of the connective tissue sheath is indicated with an asterisk (**g'**). Scale bars: 500 µm, magnified areas: 100 µm. **h-l'** Bar plots of mean fluorescence intensity (upper panels) and integrated fluorescence intensity (sum of pixel intensities, lower panels) of Involucrin (IVL, **h-h'**), Filaggrin (FLG, **i-i'**), Desmoglein 1 (DSG1, **j-j'**), Keratin 10 (KRT10, **k-k'**) and Desmoplakin (DSP, **l-l'**) immunofluorescence staining signal quantified in back skin epidermis of control (grey) and *Spink5* cKO (red) mice. The increase in total protein levels (integrated fluorescence intensity or sum of pixel intensities) in *Spink5* cKO skin could be due to the thickening of the granular layer. **m** Images of double immunofluorescence staining of Keratin 10 (KRT10, red) and Keratin 14 (KRT14, green) in back skin sections of control and *Spink5* cKO mice. Nuclei are counterstained with DAPI (blue). Each image represents one slice from a z-stack acquired by confocal fluorescence microscope. The dermal-epidermal junction is outlined with a white dashed line. Insets are magnification of the areas denoted with a dash-line rectangle. Scale bars: 50 µm, insets: 20 µm. **m'** Quantification of KRT10 and KRT14 staining co-localization using Coloc2 tool in Fiji. Pearson correlation coefficient *r* is plotted. Quantification was performed with three different images per mouse. Data in (**b** and **c**) are presented as scatter plots, where data points correspond to individual mice. Data in (**f** and **g**) is representative of five independent experiments. The graph in (**a**) shows means (line) and scatter plot (dots), where data points correspond to the mean of measurements from individual mice. At least 3 mice per time point of each group were analyzed. Graphs in (**h-l'**, **m'**) show means (bars) and scatter plots, where data points correspond to the mean of measurements from samples of individual mice (n=5). A.U., arbitrary units. Statistical significance was determined using two-tailed non-parametric Mann-Whitney test: $*p<0.05$; ns, not significant. Control mice are *Spink5*$^{fl/fl}$ and/or *Spink5*$^{fl/-}$; *Spink5* cKO mice are *KRT14-CreERT2*$^{(Tg/0)}$/*Spink5*$^{fl/fl}$ and/or *KRT14-CreERT2*$^{(Tg/0)}$/*Spink5*$^{fl/-}$.

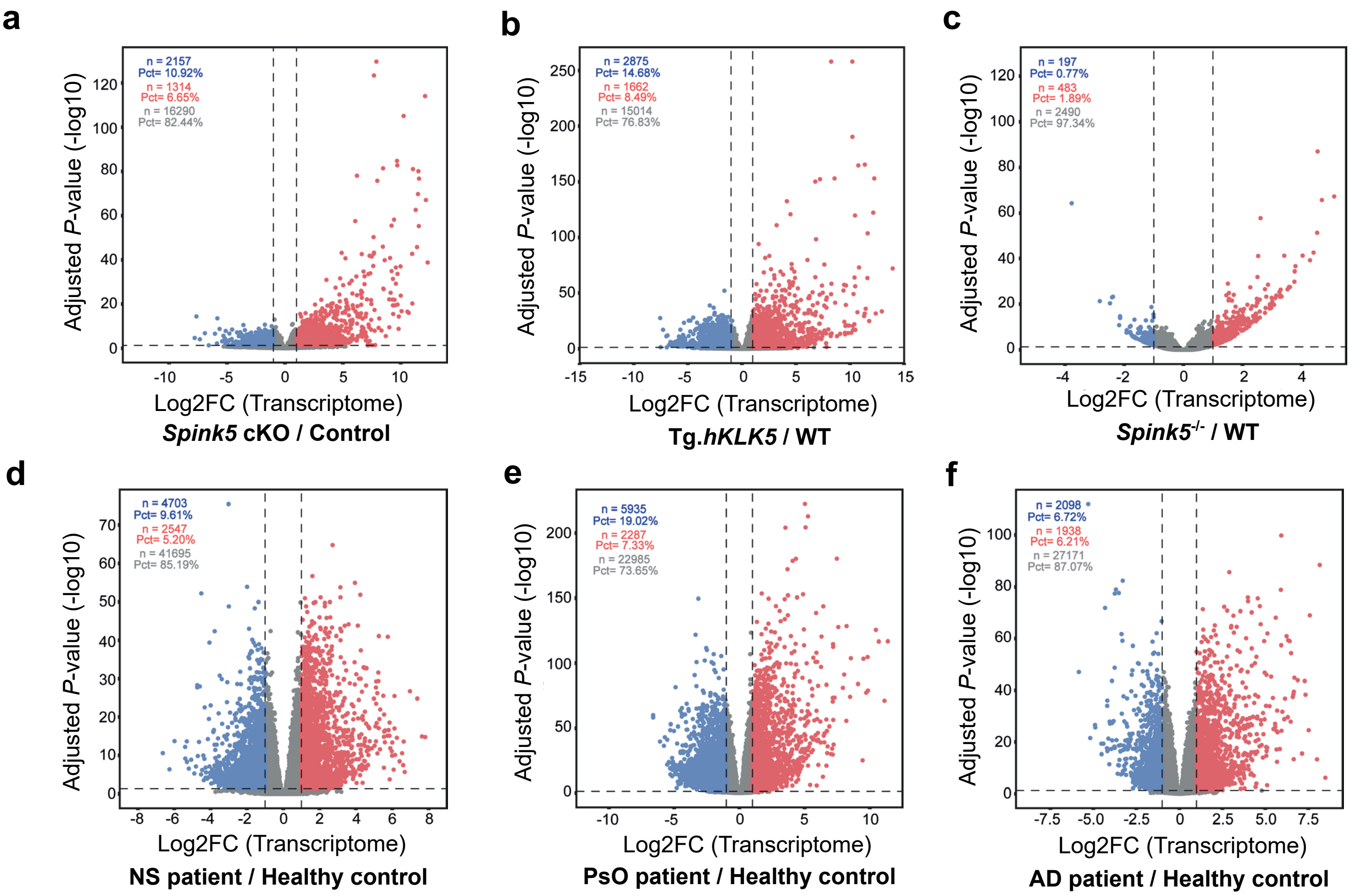

g

		Pearson correlation of transcriptomes		
	***P*-value, r-to-z Fisher's test**	NS patient vs *Spink5* cKO	NS patient vs Tg.*hKLK5*	NS patient vs *Spink5*$^{-/-}$
Pearson correlation of transcriptomes	NS patient vs *Spink5* cKO	**1**	**3.41^{-05}**	**10^{-16}**
	NS patient vs Tg.*hKLK5*	**3.41^{-05}**	**1**	**10^{-16}**
	NS patient vs *Spink5*$^{-/-}$	**10^{-16}**	**10^{-16}**	**1**

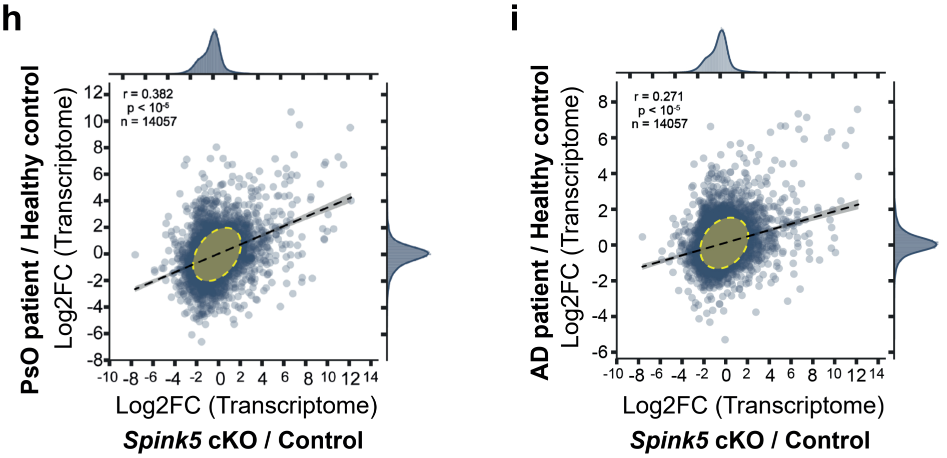

Supplementary Fig. 5: Transcriptome profiling of *Spink5* cKO, Tg.*hKLK5* and *Spink5*$^{-/-}$ mouse skin and comparison to the lesional skin transcriptomes of Netherton syndrome, psoriasis and atopic dermatitis patients.

a-f Volcano plot of differential gene expression analysis of RNAseq performed on lesional skin samples from *Spink5* cKO mice (**a**), Tg.*hKLK5* mice (**b**), *Spink5*$^{-/-}$ mice (**c**), Netherton syndrome patients (NS, **d**), psoriasis patients (PsO, **e**) and atopic dermatitis patients (AD, **f**). Data points corresponding to differentially up-regulated genes (adjusted *P*-value < 0.05 and log2 fold change > 1), differentially down-regulated genes (adjusted *P*-value < 0.05 and log2 fold change < -1) and non-significantly differentially expressed genes are colored in red, blue and grey, respectively. Horizontal and vertical dashed lines indicate the adjusted *P*-value and log2 fold change (log2FC) cutoff values, respectively. The number of non-significantly differentially expressed genes (grey), up-regulated DEGs (red) and down-regulated DEGs (blue) and their percentages are indicated in the upper left corner of each

plot. **g** Table summarizing *P*-values from r-to-z Fischer's test performed to assess the significance of the difference among the Pearson correlation coefficients from the comparisons of NS patient lesional skin transcriptome vs *Spink5* cKO, Tg.*hKLK5* or *Spink5*$^{-/-}$ skin transcriptomes shown in Figure 3a-c. **h-i** Scatter plots displaying the correlation between genes expressed in lesional skin of *Spink5* cKO mice and lesional skin of psoriasis patients (**h**) and atopic dermatitis patients (**i**). Each dot corresponds to a gene pair. The x-axes show log2 fold change of gene expression values from lesional skin of *Spink5* cKO mice as compared to the corresponding control mice. The y-axes show log2 fold change of gene expression values from lesional skin of psoriasis (**h**) or atopic dermatitis (**i**) patients as compared to healthy control subjects. The dashed black line represents the regression line with grey shaded areas indicating 95% confidence intervals. The yellow shaded region indicates the location of 95% of all data points. Pearson correlation coefficient *r*, *P*-value and the total number of data points are indicated in the upper left corner of each plot.

+

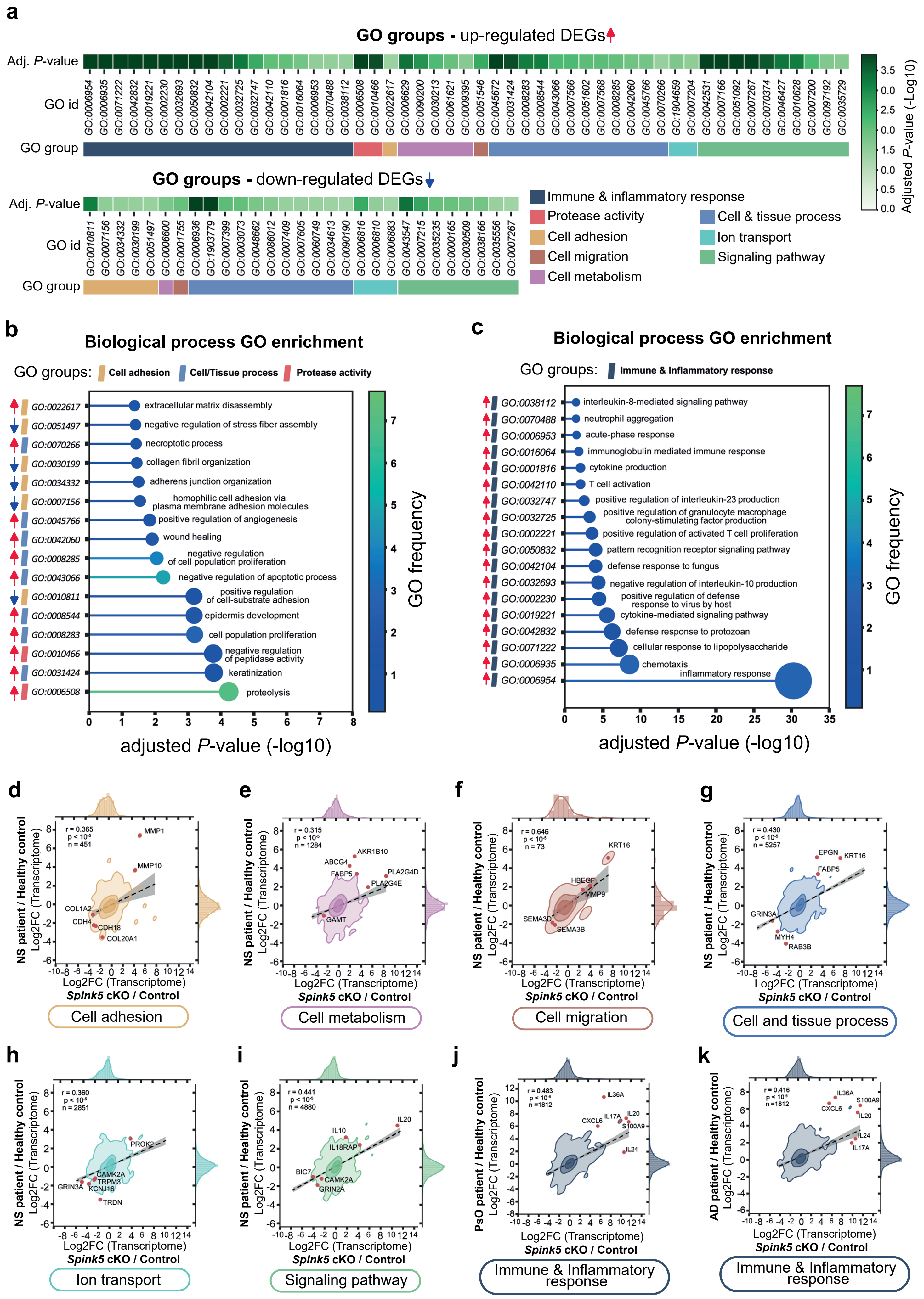
a
GO groups - up-regulated DEGs
Adj. P-value
GO id
GO group
GO groups - down-regulated DEGs
Immune & inflammatory response
Protease activity
Cell adhesion
Cell migration
Cell metabolism
Cell & tissue process
Ion transport
Signaling pathway
Adjusted P-value (-Log10)
b
Biological process GO enrichment
GO groups: Cell adhesion Cell/Tissue process Protease activity
extracellular matrix disassembly
negative regulation of stress fiber assembly
necroptotic process
collagen fibril organization
adherens junction organization
homophilic cell adhesion via plasma membrane adhesion molecules
positive regulation of angiogenesis
wound healing
negative regulation of cell population proliferation
negative regulation of apoptotic process
positive regulation of cell-substrate adhesion
epidermis development
cell population proliferation
negative regulation of peptidase activity
keratinization
proteolysis
GO frequency
adjusted P-value (-log10)
c
Biological process GO enrichment
GO groups: Immune & Inflammatory response
interleukin-8-mediated signaling pathway
neutrophil aggregation
acute-phase response
immunoglobulin mediated immune response
cytokine production
T cell activation
positive regulation of interleukin-23 production
positive regulation of granulocyte macrophage colony-stimulating factor production
positive regulation of activated T cell proliferation
pattern recognition receptor signaling pathway
defense response to fungus
negative regulation of interleukin-10 production
positive regulation of defense response to virus by host
cytokine-mediated signaling pathway
defense response to protozoan
cellular response to lipopolysaccharide
chemotaxis
inflammatory response
GO frequency
adjusted P-value (-log10)
d e f g
NS patient / Healthy control Log2FC (Transcriptome)
Log2FC (Transcriptome) Spink5 cKO / Control
Cell adhesion
Cell metabolism
Cell migration
Cell and tissue process
h i j k
PsO patient / Healthy control Log2FC (Transcriptome)
AD patient / Healthy control Log2FC (Transcriptome)
Ion transport
Signaling pathway
Immune & Inflammatory response
Immune & Inflammatory response

Supplementary Fig. 6: Gene ontology enrichment analyses of differentially expressed genes shared in the skin of *Spink5* cKO mice and lesional skin of NS, psoriasis and atopic dermatitis patients.

a Heatmap of adjusted *P*-values (-log10) of biological process gene ontology (GO) terms significantly enriched in up-regulated (red arrows) and down-regulated (blue arrows) differentially expressed genes (DEGs) common to *Spink5* cKO skin and NS patient lesional skin. The GO terms (indicated with GO id number) were assigned to different groups (color code and legend) based on the similarity of biological processes. **b** Lollipop plot of significantly enriched biological process gene ontology (GO) terms grouped into the categories "Cell and Tissue process", "Cell adhesion" and "Protease activity" that were determined within the differentially up-regulated (red arrows) and down-regulated (blue arrows) genes common to *Spink5* cKO and NS patients' lesional skin. **c** Lollipop plot of significantly enriched biological process gene ontology (GO) terms grouped into the "Immune and Inflammatory response" category that were determined within the differentially up-regulated (red arrows) and down-regulated (blue arrows) genes common to *Spink5* cKO and NS patients' lesional skin. **d-i** 2-D density plots showing the correlation between *Spink5* cKO and NS patient lesional skin transcriptomes calculated only with the subset of genes annotated to the GO terms in the groups "Cell Adhesion" (**d**), "Cell Metabolism" (**e**), "Cell Migration" (**f**), "Cell and Tissue Process" (**g**), "Ion transport" (**h**), and "Signaling pathway" (**i**). **j-k** 2-D density plots showing the correlation between *Spink5* cKO and psoriasis patient lesional skin transcriptomes (**j**) and *Spink5* cKO and atopic dermatitis lesional skin transcriptomes (**k**) calculated only with the subset of genes belonging to the GO terms within the "Immune and Inflammatory Response" category. In (**b-c**), the x-axis represents –log10 adjusted *P*-value for GO enrichment. GO frequency is indicated with a color code (color bar on the right). The size of each circle is proportional to the GO enrichment *P*-value. The GO id is indicated on the y-axis and a color bar next to it denotes the GO group category. The red or blue arrows next to each GO id number indicate enrichment in the up-regulated or down-regulated DEGs, respectively. In (**d-k**), Pearson correlation coefficient *r*, *P*-value and the total number of data points are indicated in the upper left corner of each plot.

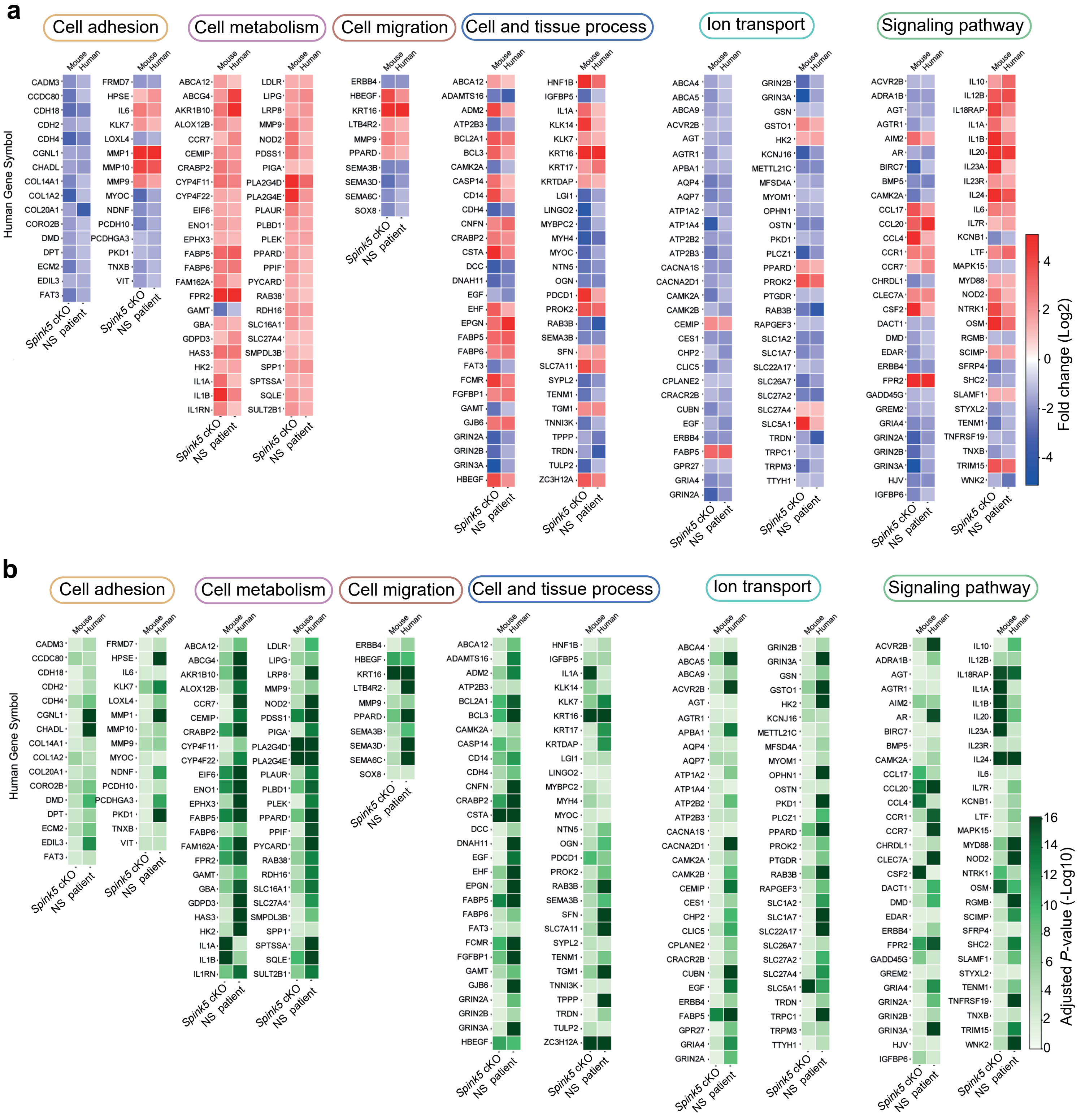

Supplementary Fig. 7: Comparative transcriptome profiling of NS patient and *Spink5* cKO mouse lesional skin – GO enrichment analyses of overlapping up-regulated and down-regulated DEGs.

a-b Heatmaps of log2 fold change values (**a**, color bar) and –log10 adjusted *P*-values (**b**, color bar) of differentially up-regulated (log2FC >1 and adj. *P*-value < 0.05) and down-regulated (log2FC < -1 and adj. *P*-value < 0.05) genes within the significantly enriched biological process GO terms identified by analyzing the differentially up-regulated and down-regulated genes common to *Spink5* cKO skin and NS patient lesional skin. For each group of significantly enriched GO terms, only selected DEGs of interest are indicated.

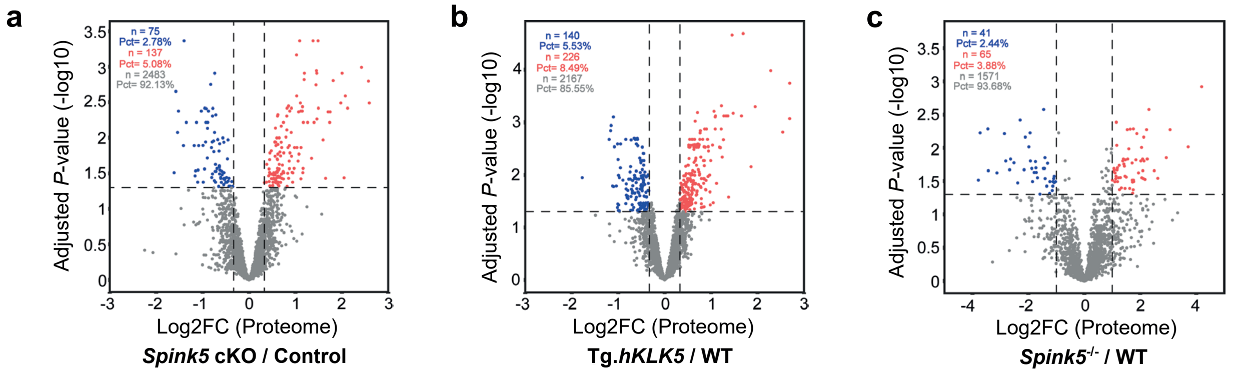

d

		Pearson correlation of proteomes		
	***P*-value, r-to-z Fisher's test**	NS patient vs *Spink5 cKO*	NS patient vs Tg.*hKLK5*	NS patient vs *Spink5*$^{-/-}$
Pearson correlation of proteomes	NS patient vs *Spink5 cKO*	**1**	**0.795946**	**0.00000022**
	NS patient vs Tg.*hKLK5*	**0.795946**	**1**	**0.00000101**
	NS patient vs *Spink5*$^{-/-}$	**0.00000022**	**0.00000101**	**1**

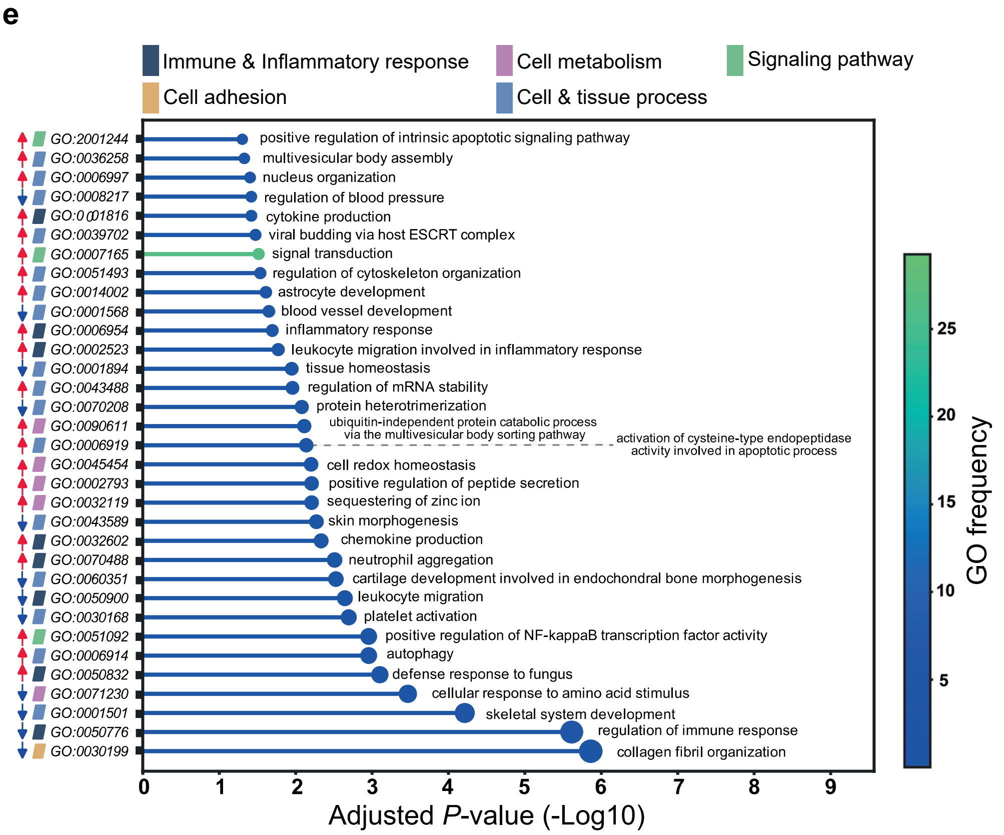

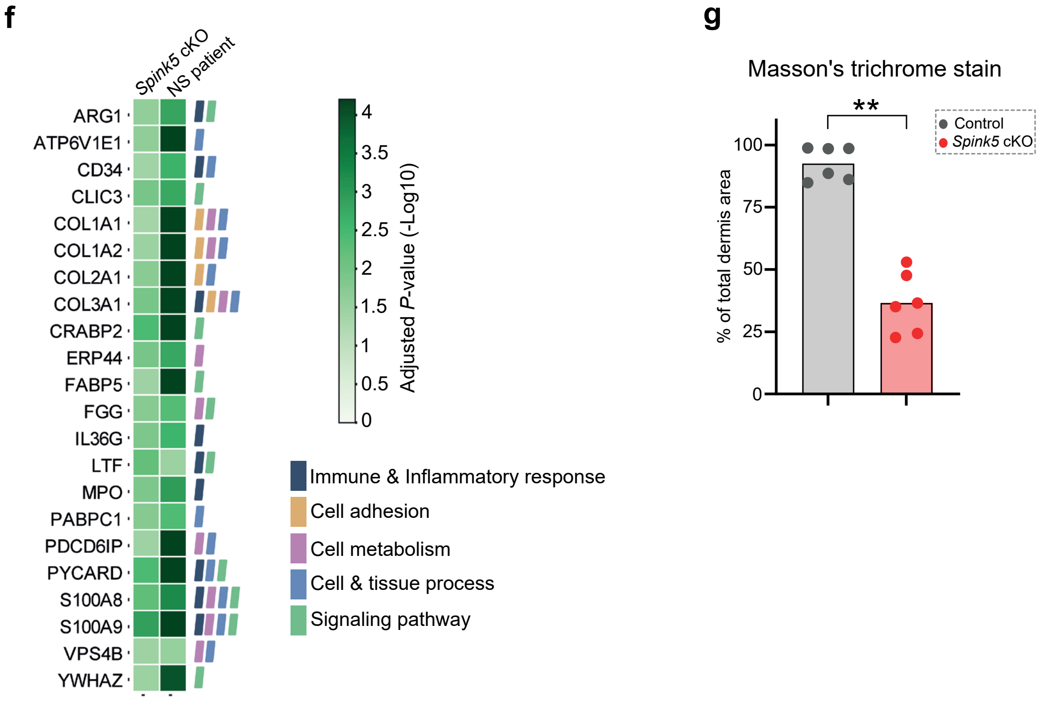

Supplementary Fig. 8: Proteome profiling of lesional skin in *Spink5* cKO, Tg.*hKLK5* and *Spink5*$^{-/-}$ mouse models.

a-c Volcano plots of differential protein expression analysis of LC-MS/MS proteomics performed on lesional skin samples from *Spink5* cKO (**a**), Tg.*hKLK5* (**b**) and *Spink5*$^{-/-}$ (**c**) mice. Data points corresponding to differentially up-regulated proteins (adjusted *P*-value < 0.05 and log2FC > 0.33 for *Spink5* cKO and Tg.*hKLK5* mouse models; adjusted *P*-value < 0.05 and log2FC > 1 for *Spink5*$^{-/-}$ mouse model), differentially down-regulated proteins (adjusted *P*-value < 0.05 and log2FC < -0.33 for *Spink5* cKO and Tg.*hKLK5* mouse models; adjusted *P*-value < 0.05 and log2FC < -1 for *Spink5*$^{-/-}$ mouse model) and non-significantly differentially expressed proteins are colored in red, blue or grey, respectively. Horizontal and vertical dashed lines indicate the adjusted *P*-value and log2 fold change (log2FC) cutoff values, respectively. The numbers of non-significantly differentially expressed proteins (DEPs, grey), up-regulated DEPs (red) and down-regulated DEPs (blue) and their percentages are indicated in the upper left corner of each plot. The log2 fold change values for the *Spink5* cKO and Tg.*hKLK5* datasets were set to 0.33 (or -0.33) to adjust for the compression of ratios that occurs in the TMT proteomics method as compared to the label-free proteomics method that was used for the *Spink5*$^{-/-}$ mouse model and NS patient lesional skin samples. **d** Table summarizing *P*-values from r-to-z Fischer's test performed to assess the significance of the difference among the Pearson correlation coefficients from the comparisons of NS patient lesional skin proteome vs *Spink5* cKO, Tg.*hKLK5* or *Spink5*$^{-/-}$ skin proteomes shown in Figure 4a-c. **e** Lollipop plot of the significantly enriched biological process GO terms within the differentially up-regulated and down-regulated proteins shared between *Spink5* cKO skin and NS patients' lesional skin. The x-axis represents –log10 adjusted *P*-value for GO enrichment. The size of each circle is proportional to the GO *P*-value. GO frequency is indicated with a color code (color bar on the right side of the plot). The color legend denotes the GO groups according to the similarity of the biological process of each GO. The red or blue arrows next to each GO id number indicate enrichment in the up-regulated or down-regulated DEPs, respectively. **f** Heatmap of –log10 *P*-values (color bar) of differentially expressed proteins within the significantly enriched biological process GO terms determined by analyzing the differentially up-regulated and down-regulated proteins common to *Spink5* cKO and NS patient lesional skin. The color legend denotes the GO groups according to the similarity of the biological process of each GO. **g** Plot of Masson's trichrome staining quantification of images shown in Figure 4f. The area of blue-stained regions is expressed as percent of total dermis area in each image. One image at 10x magnification was quantified per skin sample. The graph shows means (bars) and scatter plots, where data points correspond to measurements from samples of individual mice (n=6 mice per group). Statistical significance was determined using two-tailed non-parametric Mann-Whitney test: **p<0.01. Control mice are *Spink5*$^{fl/fl}$ and/or *Spink5*$^{fl/-}$; *Spink5* cKO mice are *KRT14-CreERT2*$^{(Tg/0)}$/*Spink5*$^{fl/fl}$ and/or *KRT14-CreERT2*$^{(Tg/0)}$/*Spink5*$^{fl/-}$.

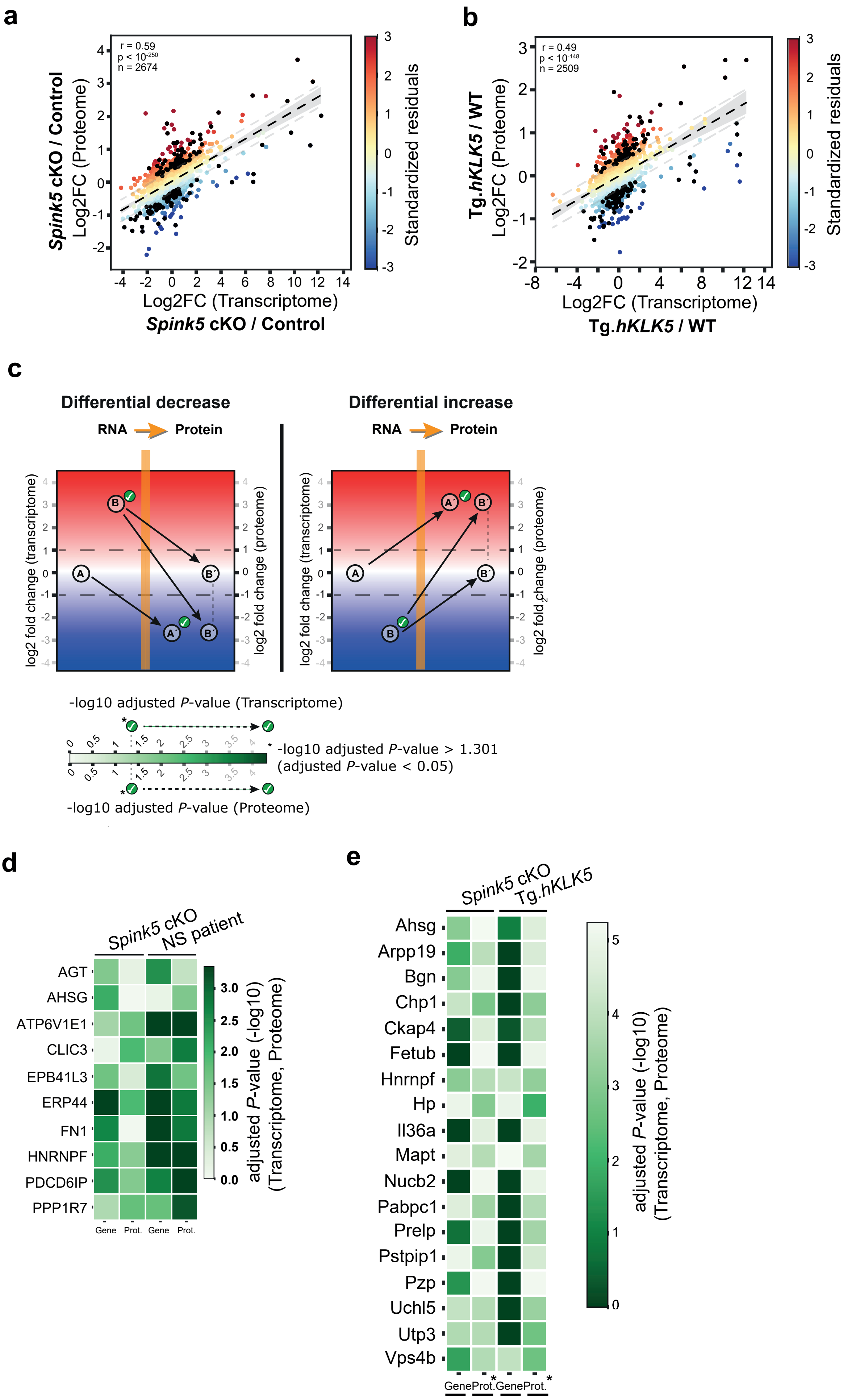

a
r = 0.59
p < 10-250
n = 2674
Spink5 cKO / Control
Log2FC (Proteome)
Log2FC (Transcriptome)
Spink5 cKO / Control
Standardized residuals
b
r = 0.49
p < 10-148
n = 2509
Tg.hKLK5 / WT
Log2FC (Proteome)
Log2FC (Transcriptome)
Tg.hKLK5 / WT
Standardized residuals
c
Differential decrease
RNA
Protein
Differential increase
RNA
Protein
log2 fold change (transcriptome)
log2 fold change (proteome)
-log10 adjusted P-value (Transcriptome)
* -log10 adjusted P-value > 1.301
(adjusted P-value < 0.05)
-log10 adjusted P-value (Proteome)
d
Spink5 cKO
NS patient
AGT
AHSG
ATP6V1E1
CLIC3
EPB41L3
ERP44
FN1
HNRNPF
PDCD6IP
PPP1R7
Gene
Prot.
Gene
Prot.
adjusted P-value (-log10)
(Transcriptome, Proteome)
e
Spink5 cKO
Tg.hKLK5
Ahsg
Arpp19
Bgn
Chp1
Ckap4
Fetub
Hnrnpf
Hp
Il36a
Mapt
Nucb2
Pabpc1
Prelp
Pstpip1
Pzp
Uchl5
Utp3
Vps4b
Gene
Prot.
Gene
Prot.
adjusted P-value (-log10)
(Transcriptome, Proteome)

Supplementary Fig. 9: Comparative analyses *Spink5* cKO, Tg.*hKLK5* and NS patient lesional skin transcriptomes and proteomes.

a-b Scatter plot of correlation between mRNA and protein fold changes in *Spink5* cKO (**a**) and Tg.*hKLK5* (**b**) lesional skin. Standardized residual values are superimposed on the scatter plots as a color gradient (color bar) and indicate the difference (standardized residual) between the observed protein fold change of each mRNA-protein pair and the expected protein fold change given by the correlation between mRNA-protein pairs. The regression line is represented as a black dashed line. Grey dashed lines mark the threshold values +1 and -1 of standardized residuals used as filter criteria for subsequent analyses. Black dots indicate the position of the selected mRNA-protein pairs common to *Spink5* cKO and Tg.*hKLK5* transcriptome-proteome datasets that belong to any significantly enriched GO term and whose standardized residual value is >1 in both *Spink5* cKO and Tg.*hKLK5* or <-1 in both *Spink5* cKO and Tg.*hKLK5* datasets. Pearson correlation coefficient *r*, *P*-value and the total number of data points are indicated in each plot. **c** Scheme depicting the filter criteria applied to the mRNA-protein pairs that belong to significantly enriched GO terms shown in Supplementary Figure 10 to obtain a list of mRNA-protein pairs (shown in Fig.5c-d and in panels **d-e** of this figure), whose protein levels are significantly shifted from the corresponding transcript expression levels in both *Spink5* cKO and NS patient skin or in both *Spink5* cKO skin and Tg.*hKLK5* mouse skin. To select genes whose protein expression level decreases (differential decrease, left panel), either of the following criteria had to be satisfied for *Spink5* cKO mice and NS patient (or Tg.*hKLK5* mice): (1) genes whose mRNA level log2 fold change is > 1 and adjusted *P*-value is < 0.05 that show a protein log2 fold change < 1 for NS patients and < 0.33 for *Spink5* cKO mice (or Tg.*hKLK5* mice) or (2) genes whose mRNA log2FC > -1 and whose protein log2 fold change < -1 for NS patients and < -0.33 for *Spink5* cKO mice (or Tg.*hKLK5* mice) and adjusted *P*-value < 0.05. To select genes whose protein expression level increases (differential increase, right panel), either of the following criteria had to be satisfied for *Spink5* cKO mice and NS patient, or alternatively Tg.*hKLK5* mice: (1) genes whose mRNA level log2 fold change is < -1 and adjusted *P*-value is < 0.05 that show a protein log2 fold change > -1 for NS patients and > -0.33 for *Spink5* cKO mice (or Tg.*hKLK5* mice), or (2) genes whose mRNA log2 fold change is < 1 and whose protein log2 fold change is > 1 for NS patients and > 0.33 for *Spink5* cKO mice (or Tg.*hKLK5* mice) and adjusted *P*-value < 0.05. **d-e** Heatmap of –log10 adjusted *P*-values of mRNA-protein pairs, whose protein levels are significantly shifted from the corresponding transcript expression levels in both NS patient and *Spink5* cKO mouse skin (**d**) or in both *Spink5* cKO and Tg.*hKLK5* mouse skin (**e**) according to the filter criteria described in (**c**).

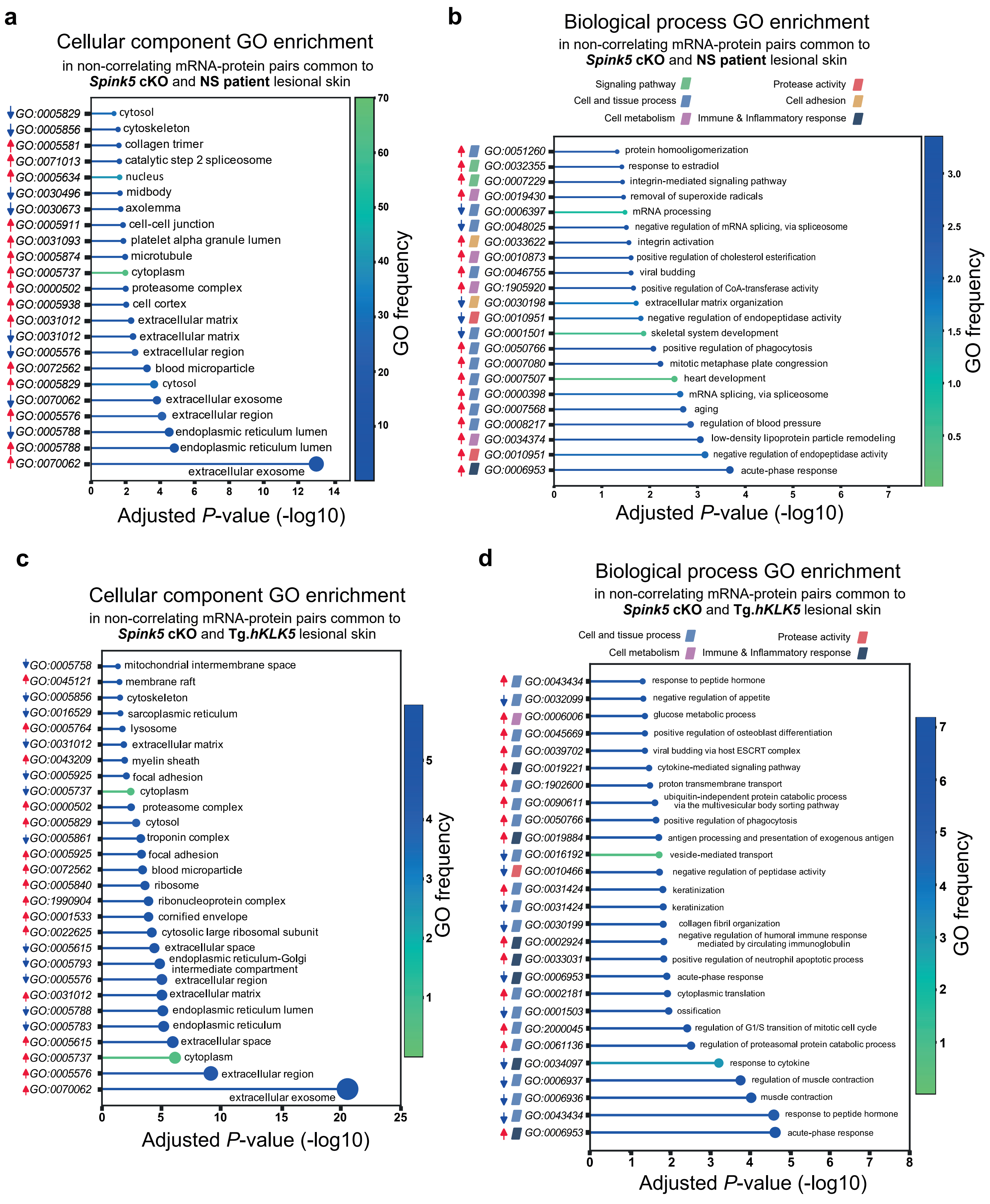

Supplementary Fig. 10: Comparative analyses of *Spink5* cKO, Tg.*hKLK5* and NS patient lesional skin transcriptomes and proteomes - GO enrichment analyses.

a-d Lollipop plots of significantly enriched cellular component (**a**, **c**) and biological process (**b**, **d**) GO terms in the set of genes common to *Spink5* cKO skin and NS patient skin (**a**, **b**) or common to *Spink5* cKO skin and Tg.*hKLK5* skin (**c**, **d**), whose mRNA-protein expression fold changes correlate poorly (standardized residuals value >1 or < -1 in both *Spink5* cKO and NS patient or in both *Spink5* cKO and Tg.*hKLK5* data sets). GO terms enriched in proteins, whose expression level is higher (up) or lower (down) than the expected from the correlation of mRNA-protein are indicated with red (up) or blue (down) arrows next to each GO id number. GO frequency is indicated with a color code (color bar on the right). The size of each circle is proportional to the GO *P*-value.

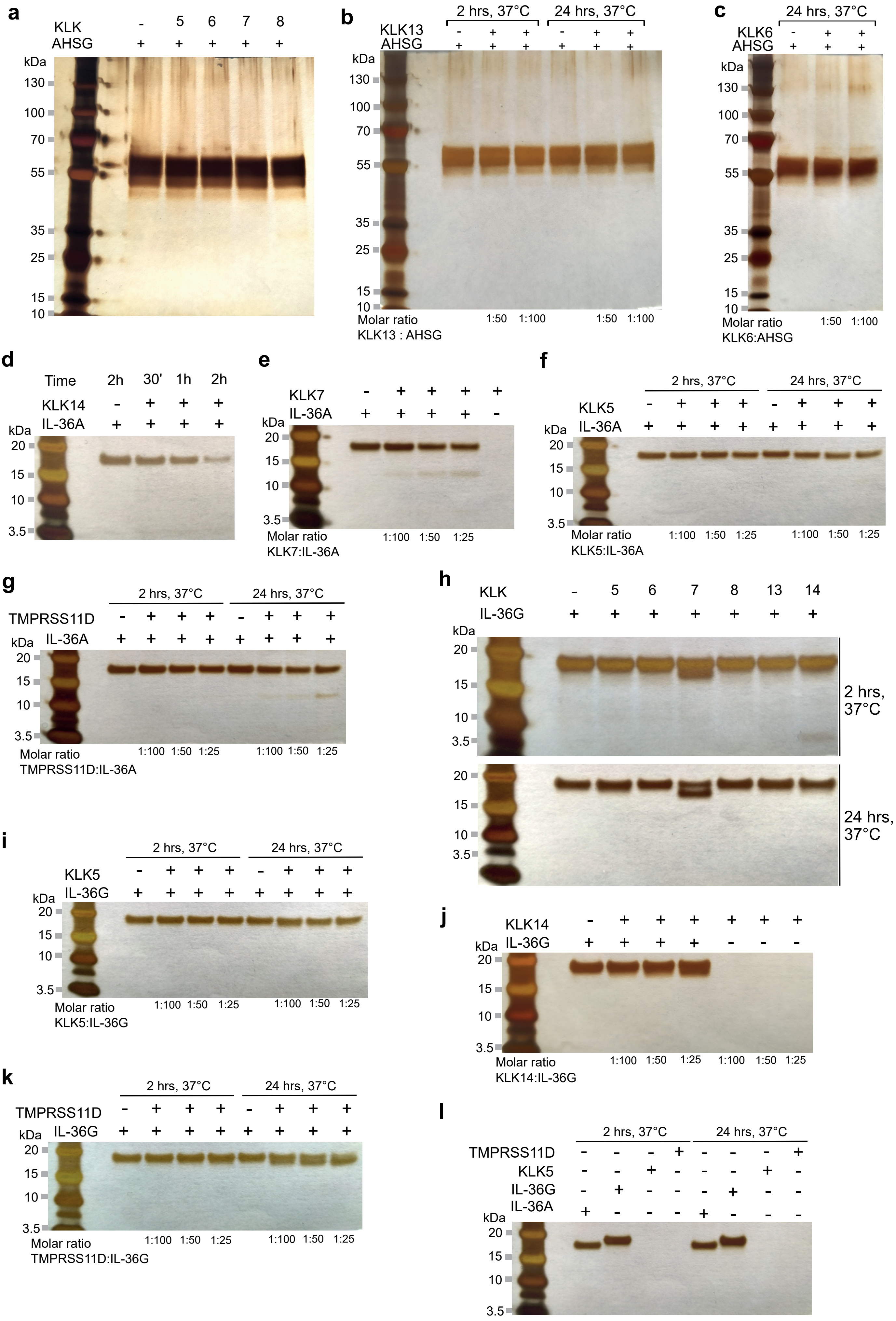
a
KLK - 5 6 7 8
AHSG + + + + +
kDa 130 100 70 55 35 25 15 10
b
2 hrs, 37°C
24 hrs, 37°C
KLK13 - + + - + +
AHSG + + + + + +
kDa 130 100 70 55 35 25 15 10
Molar ratio
KLK13 : AHSG
1:50 1:100 1:50 1:100
c
24 hrs, 37°C
KLK6 - + +
AHSG + + +
kDa 130 100 70 55 35 25 15 10
Molar ratio
KLK6:AHSG
1:50 1:100
d
Time 2h 30' 1h 2h
KLK14 - + + +
IL-36A + + + +
kDa 20 15 10 3.5
e
KLK7 - + + + +
IL-36A + + + + -
kDa 20 15 10 3.5
Molar ratio
KLK7:IL-36A
1:100 1:50 1:25
f
2 hrs, 37°C
24 hrs, 37°C
KLK5 - + + + - + + +
IL-36A + + + + + + + +
kDa 20 15 10 3.5
Molar ratio
KLK5:IL-36A
1:100 1:50 1:25 1:100 1:50 1:25
g
2 hrs, 37°C
24 hrs, 37°C
TMPRSS11D - + + + - + + +
IL-36A + + + + + + + +
kDa 20 15 10 3.5
Molar ratio
TMPRSS11D:IL-36A
1:100 1:50 1:25 1:100 1:50 1:25
h
KLK - 5 6 7 8 13 14
IL-36G + + + + + + +
kDa 20 15 10 3.5
2 hrs, 37°C
24 hrs, 37°C
i
2 hrs, 37°C
24 hrs, 37°C
KLK5 - + + + - + + +
IL-36G + + + + + + + +
kDa 20 15 10 3.5
Molar ratio
KLK5:IL-36G
1:100 1:50 1:25 1:100 1:50 1:25
j
KLK14 - + + + + + +
IL-36G + + + + - - -
kDa 20 15 10 3.5
Molar ratio
KLK14:IL-36G
1:100 1:50 1:25 1:100 1:50 1:25
k
2 hrs, 37°C
24 hrs, 37°C
TMPRSS11D - + + + - + + +
IL-36G + + + + + + + +
kDa 20 15 10 3.5
Molar ratio
TMPRSS11D:IL-36G
1:100 1:50 1:25 1:100 1:50 1:25
l
2 hrs, 37°C
24 hrs, 37°C
TMPRSS11D - - - + - - - +
KLK5 - - + - - - + -
IL-36G - + - - - + - -
IL-36A + - - - + - - -
kDa 20 15 10 3.5

Supplementary Fig. 11: Validation of protease substrates by in vitro digestion assay.
Images of silver-stained SDS-PAGE gel analyses of protein fragments obtained by in vitro digestion of recombinant human full-length AHSG (**a**-**c**), recombinant human full-length IL-36A (**d**-**g**, **l**), and recombinant human full-length IL-36G (**h**-**l**). **a** AHSG was incubated with KLK5, KLK6, KLK7, or KLK8 at enzyme:substrate molar ratio of 1:100 for 2 hours at 37°C. **b**-**c** AHSG was incubated with KLK13 (**b**) or KLK6 (**c**) at enzyme:substrate molar ratio of 1:100 and 1:50 for 2 and/or 24 hours at 37°C. Cleavage of AHSG by KLK6 and KLK13 was additionally tested at incubation time of 24 hours, since the activity of these enzymes was lower as compared to the rest of the KLKs tested. **d** Digestion of IL-36A by KLK14 at enzyme:substrate molar ratio of 1:100 was analyzed after 30-minute, 1-hour and 2-hour incubation at 37°C. **e** Digestion of IL-36A by KLK7 at enzyme:substrate molar ratio of 1:100, 1:50 and 1:25 was analyzed after 24-hour incubation at 37°C. **f**-**g** Digestion of IL-36A by KLK5 (**f**) or TMPRSS11D (**g**) at enzyme:substrate molar ratio of 1:100, 1:50 and 1:25 was analyzed after 2-hour and 24-hour incubation at 37°C. **h** IL-36G was incubated with KLK5, KLK6, KLK7, KLK8, KLK13 or KLK14 at enzyme:substrate molar ratio of 1:100 and the digestion products were analyzed after 2-hour and 24-hour incubation at 37°C. **i** Digestion of IL-36G by KLK5 at enzyme:substrate molar ratio of 1:100, 1:50 and 1:25 was analyzed after 2-hour and 24-hour incubation at 37°C. **j** Digestion of IL-36G by KLK14 at enzyme:substrate molar ratio of 1:100, 1:50 and 1:25 was analyzed after 24-hour incubation at 37°C. Reactions containing enzyme only were analyzed in parallel as negative controls. **k** Digestion of IL-36G by TMPRSS11D at enzyme:substrate molar ratio of 1:100, 1:50 and 1:25 was analyzed after 2-hour and 24-hour incubation at 37°C. **l** Negative control digestion reactions containing only substrate (IL-36A or IL-36G) or enzyme (KLK5 or TMPRSS11D) were performed in parallel and analyzed after 2-hour and 24-hour incubation at 37°C. In all panels (**a**-**l**), negative control digestion reactions containing only substrate (AHSG, IL-36A or IL-36G) were analyzed in parallel. In all panels, data are representative of two independent experiments with similar results. See also Supplementary Fig.12.

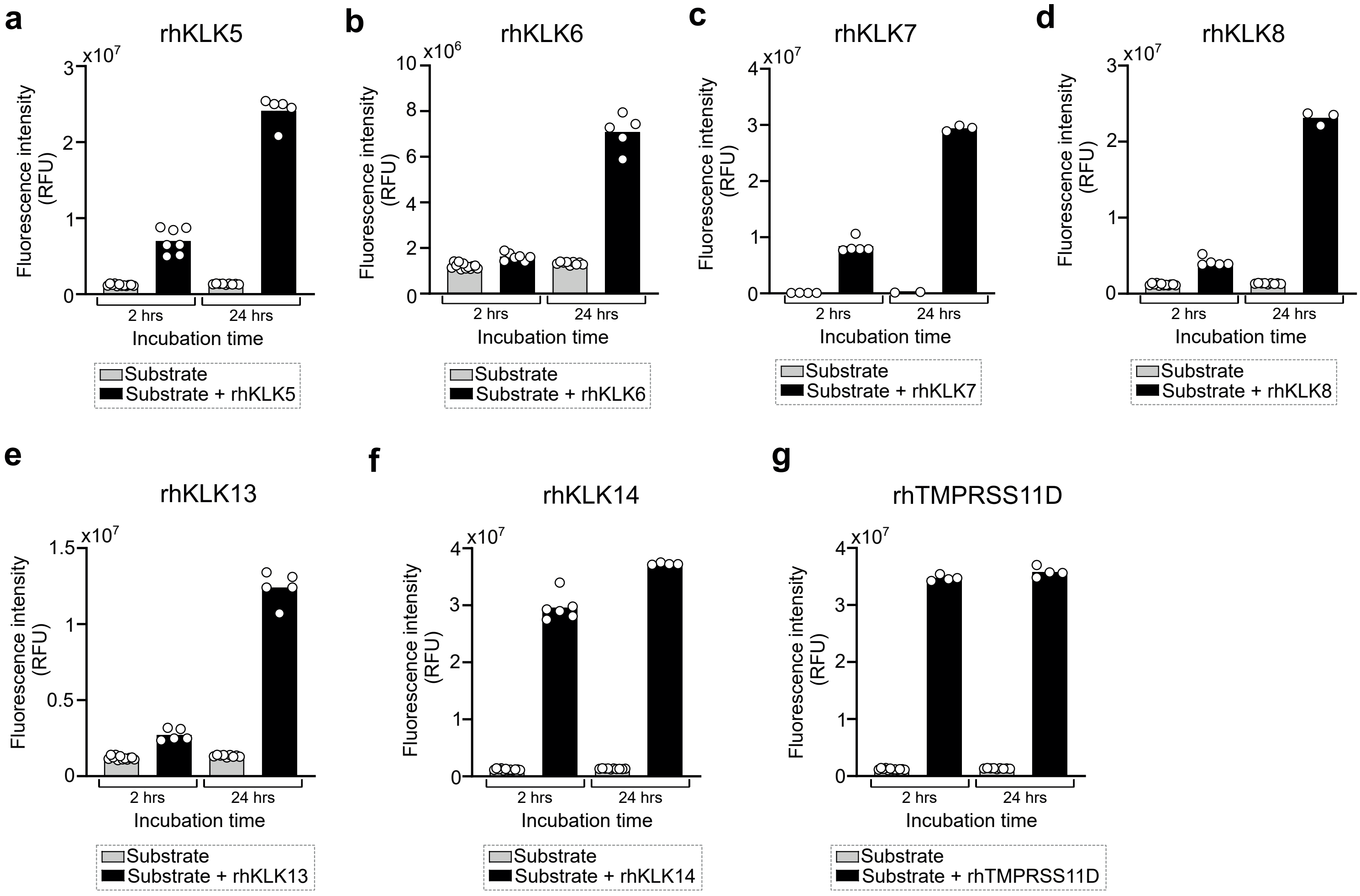

Supplementary Fig. 12: Validation of the proteolytic activity of recombinant human enzymes used in in vitro digestion assays.

a-g The activity of the recombinant human enzymes used in the in vitro digestion assays with AHSG, IL-36A or IL-36G shown in Fig.5e-f and Supplementary Fig.11 was confirmed in parallel reactions using a fluorescently labelled peptide substrate Boc-VPR-amc (for KLK5, KLK6, KLK8, KLK13, KLK14 and TMPRSS11D) or KHLY-amc (for KLK7). The same working solution (corresponding to enzyme concentration in reactions at 1:100 enzyme to substrate molar ratio, 1.6 nM final concentration of enzyme in reaction) was used for both the in vitro digestion of IL-36A, IL-36G or AHSG shown in Fig.5e-f and Supplementary Fig. 11 and the fluorogenic peptide substrate cleavage assay shown here. Fluorescence intensity was measured after 2-hour and 24-hour incubation at 37°C. Reactions containing substrate only were analyzed in parallel as a negative control. All graphs show means (bars) and scatter plots, where data points correspond to the mean of measurements from at least two independent experiments.

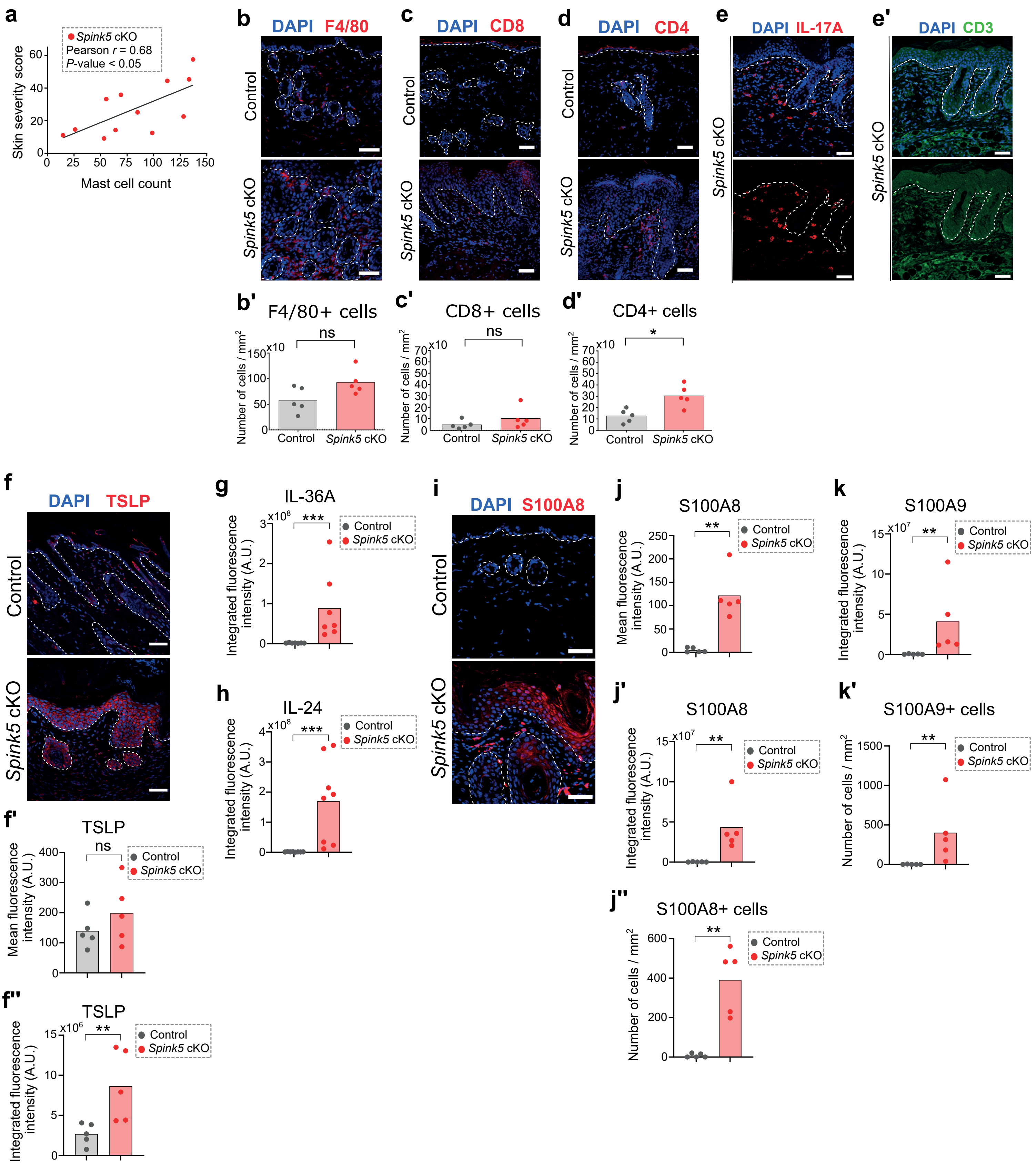

Supplementary Fig. 13: Skin inflammation phenotype of *Spink5* cKO mice.

a Scatter plot displaying the correlation of mast cell counts and whole-body skin lesion severity score measured in *Spink5* cKO mice. Regression line, Pearson correlation coefficient *r* and *P*-value are indicated. **b-b'** Immunofluorescence staining of F4/80 (marker of macrophages) (red) (**b**) in back skin cryosections of control (upper panel) and *Spink5* cKO (lower panel) mice and quantification of F4/80 marker-positive cells in immunostained skin sections (**b'**). Scale bars: 50 µm. **c-c'** Immunofluorescence staining of CD8 cell marker (red) (**c**) in back skin cryosections of control (upper panel) and *Spink5* cKO (lower panel) mice and quantification of CD8 marker-positive cells in immunostained skin sections (**c'**). Scale bars: 50 µm. **d-d'** Immunofluorescence staining of CD4 cell marker (red) (**d**) in back skin cryosections of control (upper panel) and *Spink5* cKO (lower panel) mice and quantification of CD4 marker-positive cells in immunostained skin sections (**d'**). Scale bars: 50 µm. **e-e'** Immunofluorescence staining of IL-17A (red) (**e**) and CD3 (green) (**e'**) in consecutive paraffin sections from the

back skin of a *Spink5* cKO mouse. Scale bars: 50 µm. **f-f"** Immunofluorescence staining of TSLP (**f**) in back skin paraffin sections of control (upper panel) and *Spink5* cKO (lower panel) mice and quantification of TSLP staining (red) intensity in the epidermis (**f'-f"**). Scale bars: 50 µm. **g-h** Quantification of integrated fluorescence intensity in immunofluorescence staining images of IL-36A (**g**) and IL-24 (**h**). **i** Immunofluorescence staining of S100A8 (red) in back skin paraffin sections of control (upper panel) and *Spink5* cKO (lower panel) mice. Scale bars: 50 µm. **j-j"** Quantification of mean fluorescence intensity (**j**) and integrated fluorescence intensity (**j'**) of S100A8 immunostaining signal in epidermis and number of S100A8+ cells in dermis (**j"**) quantified in images of S100A8 immunofluorescence staining of control and *Spink5* cKO back skin. **k-k'** Quantification of integrated fluorescence intensity of S100A9 immunostaining signal in epidermis (**k**) and number of S100A9+ cells in dermis (**k'**) quantified in immunofluorescence staining images of skin paraffin sections from control and *Spink5* cKO mice. Data in (**a**) is presented as a scatter plot where each data point corresponds to individual mice (n=12). Data in (**b'**, **c'**, **d'**, **f'-h** and **j-k'**) are means (bars) and scatter plots, where data points correspond to values measured from samples of individual mice (n≥5 per group). Statistical significance was determined using two-tailed unpaired non-parametric Mann-Whitney test: *p<0.05, **p<0.01, ***p<0.001; ns, not significant. Control mice are *Spink5*$^{fl/fl}$ and/or *Spink5*$^{fl/-}$; *Spink5* cKO mice are *KRT14-CreERT2*$^{(Tg/0)}$/*Spink5*$^{fl/fl}$ and/or *KRT14-CreERT2*$^{(Tg/0)}$/*Spink5*$^{fl/-}$.

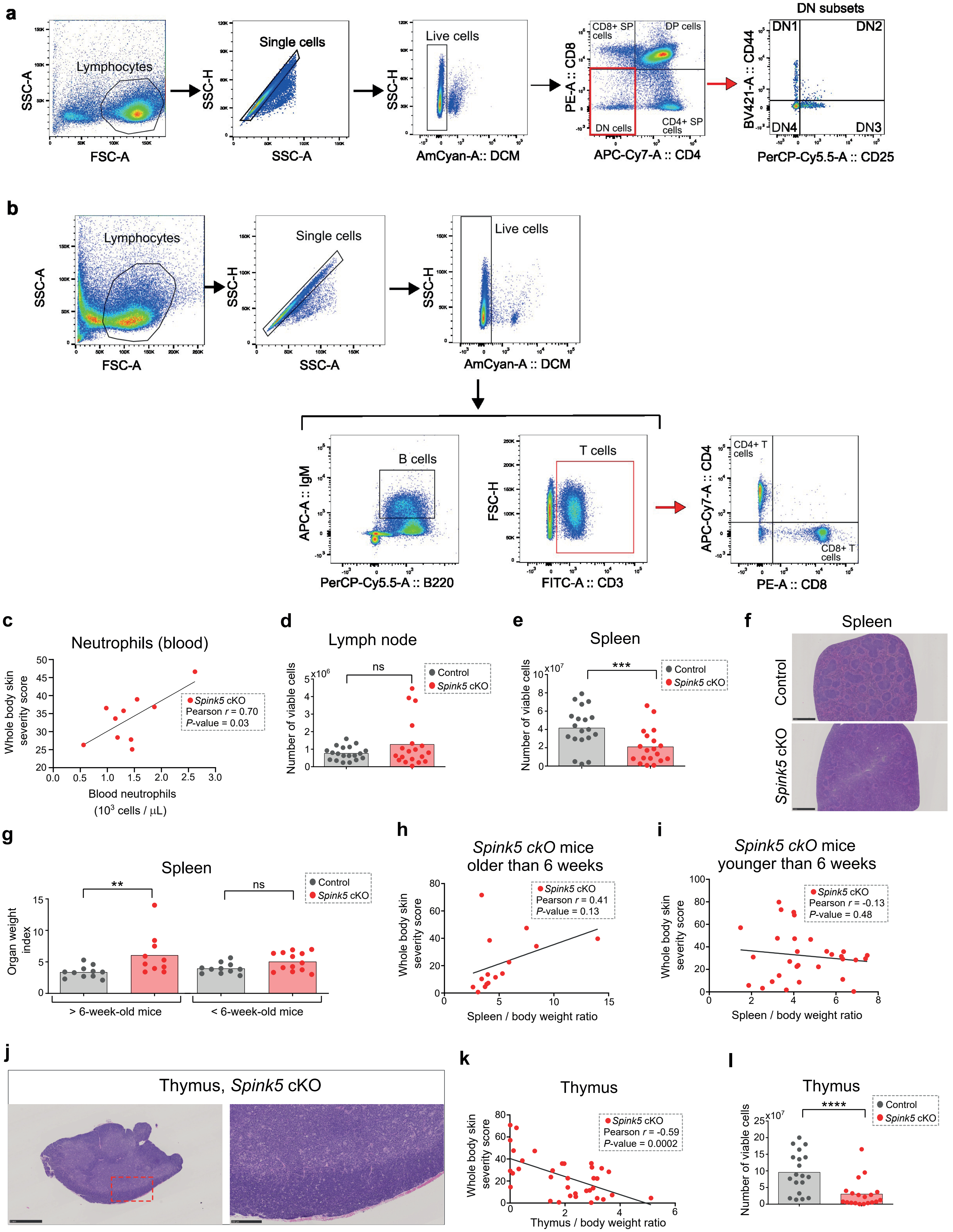

Supplementary Fig. 14: Analyses of lymphoid organs in *Spink5* cKO mice.

a Gating strategy for flow cytometry analyses of single cell suspensions from thymus. Lymphocytes (FSC-A vs SSC-A) were gated for single cells (SSC-A vs SSC-H). Single cells were further gated depending on cell viability. Cells expressing CD4 or CD8 were identified within the live cells gate (DCM-). Cells expressing CD44 and/or CD25 were identified within the CD4-CD8- double-negative (DN) cells gate. Gates are indicated with black or red

contours and selection of events from each gate is indicated with a black or red arrow, respectively. **b** Gating strategy for flow cytometry analyses of single cell suspensions from inguinal lymph node and spleen. Lymphocytes (FSC-A vs SSC-A) were gated for single cells (SSC-A vs SSC-H). Single cells were further gated depending on cell viability. Cells expressing B220 and IgM (B cells) or CD3 (T cells) were identified within the live cells gate (DCM-). CD8+ T cells and CD4+ T cells were identified within the CD3+ T cell gate. **c** Scatter plot of correlation between blood neutrophil counts and whole-body skin severity score in *Spink5* cKO mice. Regression line, Pearson correlation coefficient *r* and *P*-value are indicated. **d-e** Total number of viable cells (trypan-blue dye exclusion) recovered from inguinal lymph node (**d**) and spleen (**e**) of control (grey) and *Spink5* cKO (red) littermate mice. **f** Images of hematoxylin- and eosin-stained paraffin section of spleen from 4-week-old control (upper panel) and *Spink5* cKO (lower panel) littermate mice. Scale bar: 1 mm. **g** Spleen weight index (spleen weight in milligrams/body weight in grams) of control (grey) and *Spink5* cKO (red) littermate mice according to age groups. **h-i** Scatter plot of correlation between spleen/body weight ratio (spleen weight in milligrams/body weight in grams) and whole-body skin severity score in *Spink5* cKO mice older than 6 weeks (**h**) or younger than 6 weeks (**i**). Regression line, Pearson correlation coefficient *r* and *P*-value are indicated. **j** Images of hematoxylin- and eosin-stained paraffin section of atrophied thymus from a 4-week-old *Spink5* cKO mouse with spontaneous *Spink5* floxed allele excision. Scale bar: 1 mm, inset: 250 µm. **k** Scatter plot of correlation between thymus/body weight ratio (thymus weight in milligrams/body weight in grams) and whole-body skin severity score of *Spink5* cKO mice. Regression line, Pearson correlation coefficient *r* and *P*-value are indicated. **l** Total number of viable cells (trypan-blue dye exclusion) recovered from thymus of control (grey) and *Spink5* cKO (red) littermate mice.

In (**a-b**), gates are indicated with black or red contours and selection of events from each gate is indicated with a black or red arrow, respectively. Data in (**d-e**, **g** and **l**) are means (bars) and scatter plots, where data points correspond to values measured from samples of individual mice (n≥5 per group). Statistical significance was determined using two-tailed non-parametric Wilcoxon matched-pairs signed rank test: **p<0.01, ***p<0.001, ****p<0.0001, ns (not significant). Control mice are *Spink5*$^{fl/fl}$ and/or *Spink5*$^{fl/-}$; *Spink5* cKO mice are *KRT14-CreERT2*$^{(Tg/0)}$/*Spink5*$^{fl/fl}$ and/or *KRT14-CreERT2*$^{(Tg/0)}$/*Spink5*$^{fl/-}$.

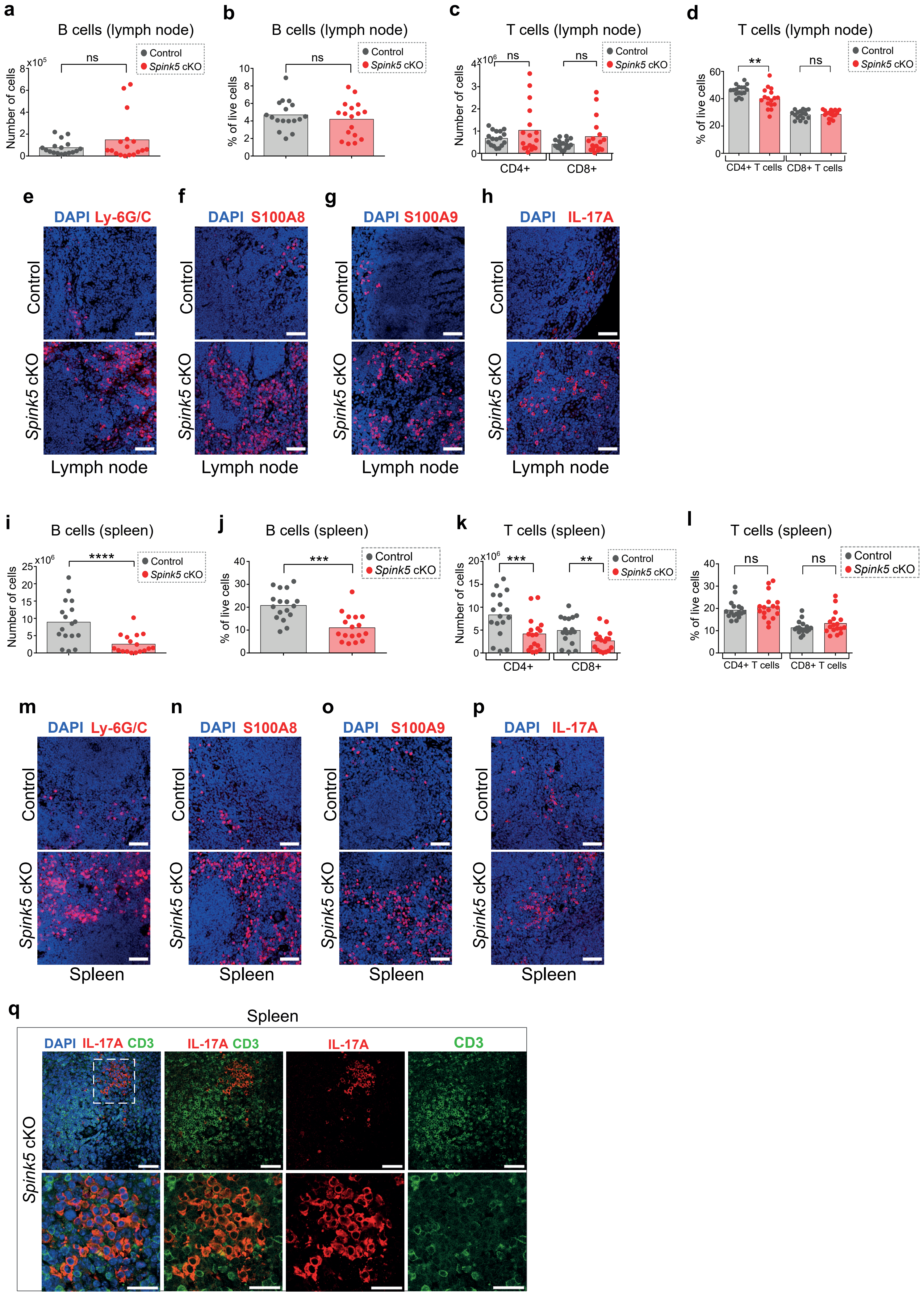
a
B cells (lymph node)
Number of cells
x10^5
ns
Control
Spink5 cKO
b
B cells (lymph node)
% of live cells
ns
Control
Spink5 cKO
c
T cells (lymph node)
Number of cells
x10^6
ns
ns
Control
Spink5 cKO
CD4+
CD8+
d
T cells (lymph node)
% of live cells
**
ns
Control
Spink5 cKO
CD4+ T cells
CD8+ T cells
e
DAPI Ly-6G/C
f
DAPI S100A8
g
DAPI S100A9
h
DAPI IL-17A
Control
Spink5 cKO
Lymph node
i
B cells (spleen)
Number of cells
x10^6
****
Control
Spink5 cKO
j
B cells (spleen)
% of live cells
***
Control
Spink5 cKO
k
T cells (spleen)
Number of cells
x10^6
***
**
Control
Spink5 cKO
CD4+
CD8+
l
T cells (spleen)
% of live cells
ns
ns
Control
Spink5 cKO
CD4+ T cells
CD8+ T cells
m
DAPI Ly-6G/C
n
DAPI S100A8
o
DAPI S100A9
p
DAPI IL-17A
Control
Spink5 cKO
Spleen
q
Spleen
DAPI IL-17A CD3
IL-17A CD3
IL-17A
CD3
Spink5 cKO

Supplementary Fig. 15: Analyses of inguinal lymph nodes and spleen in *Spink5* cKO mice.
a-b Number (**a**) and frequency (**b**) of B cells (B220+IgM+ cells) in inguinal lymph nodes of control (grey) and *Spink5* cKO littermate (red) mice determined by flow cytometry. **c-d** Number (**c**) and frequency (**d**) of CD4+ and CD8+ T cells in inguinal lymph nodes of control (grey) and *Spink5* cKO littermate (red) mice determined by flow cytometry. **e-h** Immunofluorescence staining (red) of Ly-6G/C (**e**), S100A8 (**f**), S100A9 (**g**) and IL-17A (**h**) in paraffin sections from inguinal lymph nodes of control (upper panels) and *Spink5* cKO (lower panels) mice. Scale bars: 50 µm. **i-j** Number (**i**) and frequency (**j**) of B cells (B220+IgM+ cells) in spleen of control (grey) and *Spink5* cKO littermate (red) mice determined by flow cytometry. **k-l** Number (**k**) and frequency (**l**) of CD4+ and CD8+ T cells in spleen of control (grey) and *Spink5* cKO littermate (red) mice determined by flow cytometry. **m-p** Immunofluorescence staining (red) of Ly-6G/C (**m**), S100A8 (**n**), S100A9 (**o**) and IL-17A (**p**) in paraffin sections from spleen of control (upper panels) and *Spink5* cKO (lower panels) mice. Scale bars: 50 µm. **q** Double immunofluorescence staining of CD3 and IL-17A in spleen paraffin sections from *Spink5* cKO mice. CD3 and IL-17A merged images and images of CD3 and IL-17A of the same tissue field are shown. Images in the lower panel are a magnification of the region outlined with a white dashed-line rectangle in the 3-channel merged image shown in the upper panel. Scale bars: 50 µm (upper panels), 25 µm (lower panels). Graphs in (**a-d**) and (**i-l**) show means (bars) and scatter plots, where data points correspond to values measured from samples of individual mice (n≥5 per group). Statistical significance was determined using two-tailed non-parametric Wilcoxon matched-pairs signed rank test: **p<0.01, ***p<0.001, ****p<0.0001, ns (not significant). Control mice are *Spink5*$^{fl/fl}$ and/or *Spink5*$^{fl/-}$; *Spink5* cKO mice are *KRT14-CreERT2*$^{(Tg/0)}$/*Spink5*$^{fl/fl}$ and/or *KRT14-CreERT2*$^{(Tg/0)}$/*Spink5*$^{fl/-}$. Data in (**e-h**) and (**m-q**) is representative of immunofluorescence staining performed on inguinal lymph node/spleen samples from at least five different mice.

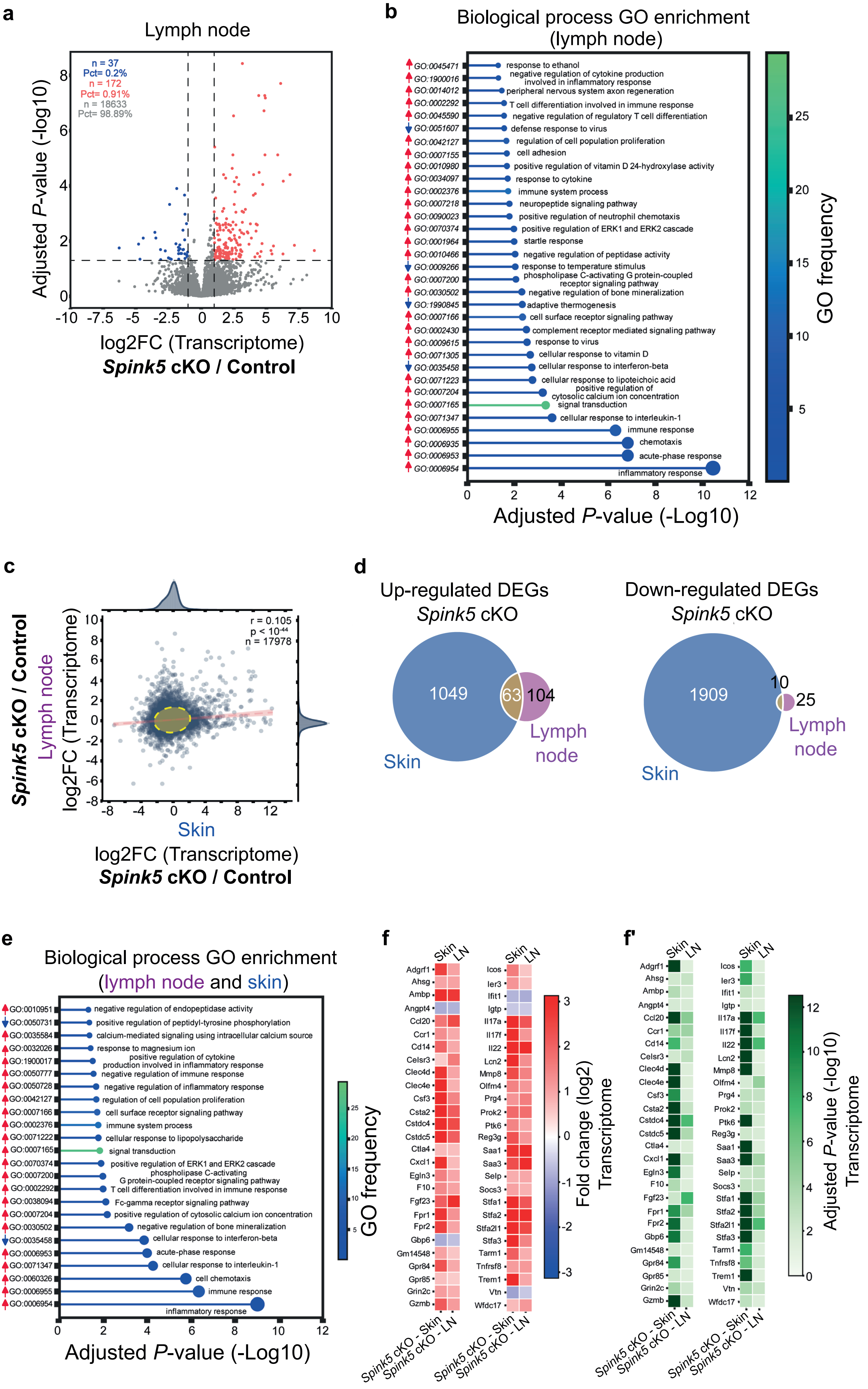
a
Lymph node
n = 37
Pct= 0.2%
n = 172
Pct= 0.91%
n = 18633
Pct= 98.89%
Adjusted P-value (-log10)
log2FC (Transcriptome)
Spink5 cKO / Control
b
Biological process GO enrichment (lymph node)
GO:0045471 response to ethanol
GO:1900016 negative regulation of cytokine production involved in inflammatory response
GO:0014012 peripheral nervous system axon regeneration
GO:0002292 T cell differentiation involved in immune response
GO:0045590 negative regulation of regulatory T cell differentiation
GO:0051607 defense response to virus
GO:0042127 regulation of cell population proliferation
GO:0007155 cell adhesion
GO:0010980 positive regulation of vitamin D 24-hydroxylase activity
GO:0034097 response to cytokine
GO:0002376 immune system process
GO:0007218 neuropeptide signaling pathway
GO:0090023 positive regulation of neutrophil chemotaxis
GO:0070374 positive regulation of ERK1 and ERK2 cascade
GO:0001964 startle response
GO:0010466 negative regulation of peptidase activity
GO:0009266 response to temperature stimulus
GO:0007200 phospholipase C-activating G protein-coupled receptor signaling pathway
GO:0030502 negative regulation of bone mineralization
GO:1990845 adaptive thermogenesis
GO:0007166 cell surface receptor signaling pathway
GO:0002430 complement receptor mediated signaling pathway
GO:0009615 response to virus
GO:0071305 cellular response to vitamin D
GO:0035458 cellular response to interferon-beta
GO:0071223 cellular response to lipoteichoic acid
GO:0007204 positive regulation of cytosolic calcium ion concentration
GO:0007165 signal transduction
GO:0071347 cellular response to interleukin-1
GO:0006955 immune response
GO:0006935 chemotaxis
GO:0006953 acute-phase response
GO:0006954 inflammatory response
Adjusted P-value (-Log10)
GO frequency
c
r = 0.105
p < 10^-44
n = 17978
Spink5 cKO / Control
Lymph node
log2FC (Transcriptome)
Skin
log2FC (Transcriptome)
Spink5 cKO / Control
d
Up-regulated DEGs
Spink5 cKO
1049
63
104
Skin
Lymph node
Down-regulated DEGs
Spink5 cKO
1909
10
25
Skin
Lymph node
e
Biological process GO enrichment (lymph node and skin)
GO:0010951 negative regulation of endopeptidase activity
GO:0050731 positive regulation of peptidyl-tyrosine phosphorylation
GO:0035584 calcium-mediated signaling using intracellular calcium source
GO:0032026 response to magnesium ion
GO:1900017 positive regulation of cytokine production involved in inflammatory response
GO:0050777 negative regulation of immune response
GO:0050728 negative regulation of inflammatory response
GO:0042127 regulation of cell population proliferation
GO:0007166 cell surface receptor signaling pathway
GO:0002376 immune system process
GO:0071222 cellular response to lipopolysaccharide
GO:0007165 signal transduction
GO:0070374 positive regulation of ERK1 and ERK2 cascade
GO:0007200 phospholipase C-activating G protein-coupled receptor signaling pathway
GO:0002292 T cell differentiation involved in immune response
GO:0038094 Fc-gamma receptor signaling pathway
GO:0007204 positive regulation of cytosolic calcium ion concentration
GO:0030502 negative regulation of bone mineralization
GO:0035458 cellular response to interferon-beta
GO:0006953 acute-phase response
GO:0071347 cellular response to interleukin-1
GO:0060326 cell chemotaxis
GO:0006955 immune response
GO:0006954 inflammatory response
Adjusted P-value (-Log10)
GO frequency
f
Skin LN
Adgrf1 Ahsg Ambp Angpt4 Ccl20 Ccr1 Cd14 Celsr3 Clec4d Clec4e Csf3 Csta2 Cstdc4 Cstdc5 Ctla4 Cxcl1 Egln3 F10 Fgf23 Fpr1 Fpr2 Gbp6 Gm14548 Gpr84 Gpr85 Grin2c Gzmb
Icos Ier3 Ifit1 Igtp Il17a Il17f Il22 Lcn2 Mmp8 Olfm4 Prg4 Prok2 Ptk6 Reg3g Saa1 Saa3 Selp Socs3 Stfa1 Stfa2 Stfa2l1 Stfa3 Tarm1 Tnfrsf8 Trem1 Vtn Wfdc17
Fold change (log2) Transcriptome
Spink5 cKO - Skin
Spink5 cKO - LN
f'
Skin LN
Adjusted P-value (-log10) Transcriptome
Spink5 cKO - Skin
Spink5 cKO - LN

Supplementary Fig. 16: Transcriptome analysis of inguinal lymph nodes in *Spink5* cKO mice.

a Volcano plot of differential gene expression analysis of RNAseq performed on inguinal lymph nodes from control and *Spink5* cKO mice. Data points corresponding to differentially up-regulated genes (adjusted *P*-value < 0.05 and log2 fold change > 1), differentially down-regulated genes (adjusted *P*-value < 0.05 and log2 fold change < -1) and non-significantly differentially expressed genes are colored in red, blue and grey, respectively. Horizontal and vertical dashed lines indicate the adjusted *P*-value and log2FC cutoff values, respectively. The number of non-significantly differentially expressed genes (grey), up-regulated DEGs (red) and down-regulated DEGs (blue) and their percentages are indicated in the upper left corner of the plot. **b** Lollipop plot of significantly enriched biological process GO terms identified in differentially up-regulated and down-regulated genes in *Spink5* cKO lymph nodes. The red or blue arrows next to each GO id number indicate enrichment in the up-regulated or down-regulated DEGs, respectively. GO frequency is indicated with a color code (color bar on the right). The size of each circle is proportional to the GO *P*-value. **c** Scatter plot displaying the correlation between differential gene expression in skin and lymph node in *Spink5* cKO mice. Each dot corresponds to a gene pair. The x-axis shows log2 fold change of gene expression values in lesional skin of *Spink5* cKO mice as compared to the corresponding control mice. The y-axis shows log2 fold change of gene expression values in inguinal lymph nodes of *Spink5* cKO mice as compared to control mice. The pink dashed line represents the regression line with pink shaded areas indicating 95% confidence intervals. The yellow shaded region indicates the location of 95% of all data points. Pearson correlation coefficient *r*, *P*-value and the total number of data points are indicated in the upper right corner of the plot. **d** Venn diagrams showing intersection of differentially up-regulated genes (adjusted *P*-value < 0.05 and log2 fold change > 1) and down-regulated genes (adjusted *P*-value < 0.05 and log2 fold change < -1) in skin (blue) and lymph nodes (purple) of *Spink5* cKO mice. **e** Lollipop plot of significantly enriched biological process GO terms identified in differentially up-regulated and down-regulated genes common to *Spink5* cKO skin and lymph nodes. The red or blue arrows next to each GO id number indicate enrichment in the up-regulated or down-regulated DEGs, respectively. GO frequency is indicated with a color code (color bar on the right). **f-f'** Heatmaps of log2 fold change values (**f**) and –log10 P-values (**f'**) of differentially up-regulated (log2FC >1 and adjusted *P*-value < 0.05) and down-regulated (log2FC < -1 and adjusted *P*-value < 0.05) genes within the significantly enriched biological process GOs terms identified by analyzing the differentially up-regulated and down-regulated genes common to *Spink5* cKO skin and lymph nodes. DEG, differentially expressed gene; GO, gene ontology; LN, lymph node.

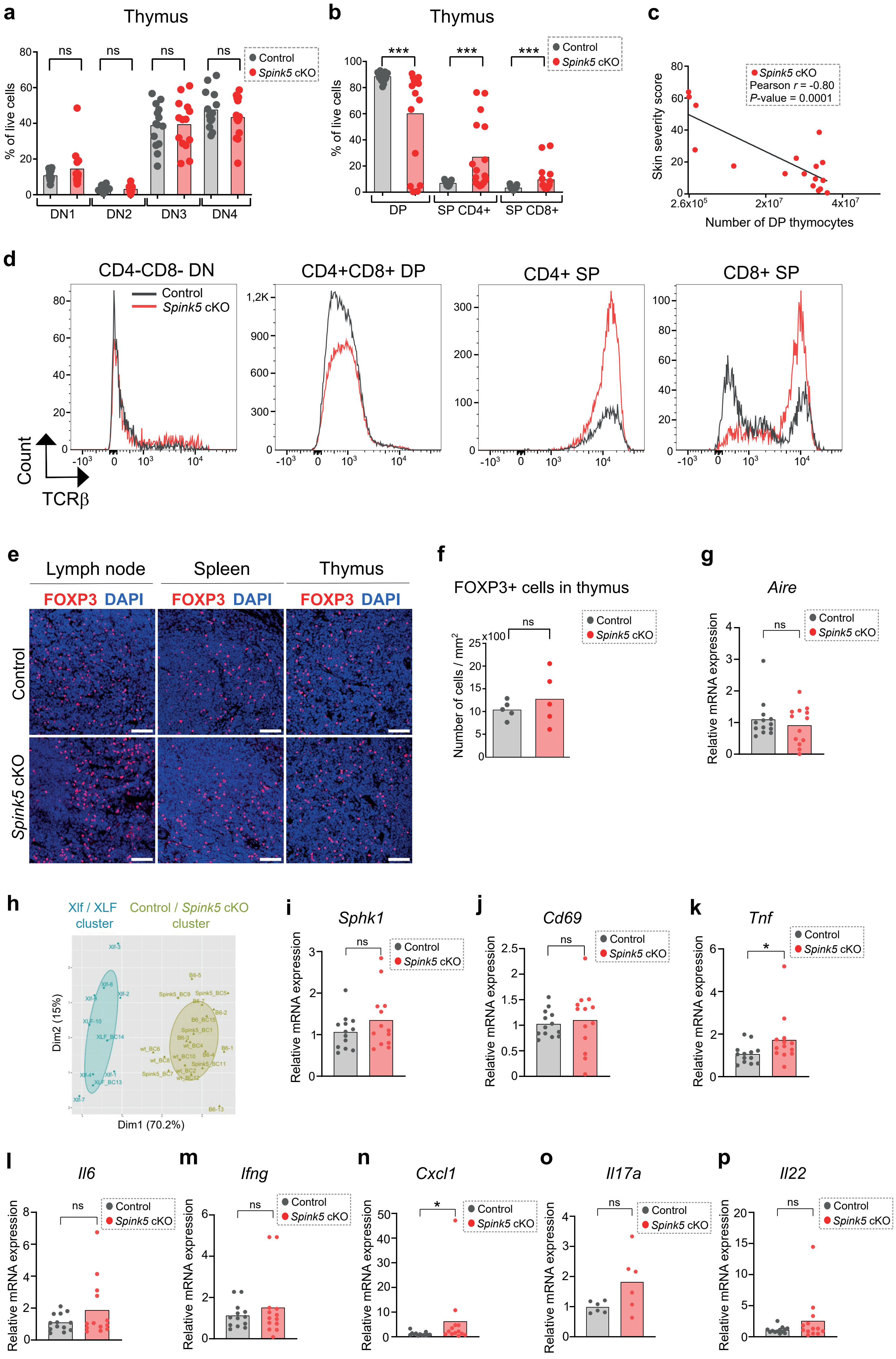
a
Thymus
% of live cells
ns
Control
Spink5 cKO
DN1
DN2
DN3
DN4
b
Thymus
% of live cells
***
DP
SP CD4+
SP CD8+
c
Spink5 cKO
Pearson r = -0.80
P-value = 0.0001
Skin severity score
Number of DP thymocytes
2.6x10^5
2x10^7
4x10^7
d
CD4-CD8- DN
CD4+CD8+ DP
CD4+ SP
CD8+ SP
Count
TCRβ
e
Lymph node
Spleen
Thymus
FOXP3 DAPI
Control
Spink5 cKO
f
FOXP3+ cells in thymus
Number of cells / mm²
x100
g
Aire
Relative mRNA expression
h
Xlf / XLF cluster
Control / Spink5 cKO cluster
Dim2 (15%)
Dim1 (70.2%)
i
Sphk1
j
Cd69
k
Tnf
*
l
Il6
m
Ifng
n
Cxcl1
o
Il17a
p
Il22

Supplementary Fig. 17: Cellular and molecular analyses of thymus development in *Spink5* cKO mice.

a Frequency of DN1 (CD25-CD44+), DN2 (CD25+CD44+), DN3 (CD25+CD44-) and DN4 (CD25-CD44-) thymocytes in the thymus of control (grey) and *Spink5* cKO (red) littermates determined by flow cytometry. **b** Frequency of DP (CD8+CD4+), SP CD4+ (single-positive CD4+CD8-) and SP CD8+ (single-positive CD4-CD8+) thymocytes in the thymus of control (grey) and *Spink5* cKO (red) littermates determined by flow cytometry. **c** Scatter plot of the correlation between the number of CD4+CD8+ double-positive (DP) thymocytes and whole-body skin severity score measured in *Spink5* cKO mice. Regression line, Pearson correlation coefficient r and P-value are indicated. **d** Overlay histograms of TCRβ cell surface expression determined by flow cytometry in different subsets of thymocytes from control (black) and *Spink5* cKO (red) littermate mice. The levels of TCRβ were progressively increasing as the thymocytes underwent DN through SP maturation, suggesting that DP thymocytes in *Spink5* cKO mice were positively selected. **e** Immunofluorescence staining of FOXP3 (red) in paraffin sections of lymph node, spleen and thymus from control (upper panels) and *Spink5* cKO (lower panels) mice. Scale bars: 50 µm. Data is representative of analyses performed with samples from five different mice of each genotype. Nuclei are counterstained with DAPI (blue). **f** Quantification of the number of FOXP3+ cells detected by immunofluorescence staining on paraffin sections of thymi from control (grey) and *Spink5* cKO mice (red). In the thymic medulla, SP thymocytes undergo negative selection resulting in the deletion of autoreactive single-positive thymocytes, maturation into CD4 or CD8 naïve T cells or differentiation into FOXP3+ T regulatory cells. Loss-of-function mutations in FOXP3 cause autoimmune diseases in humans and mice. *Spink5* cKO and control littermates displayed similar number of FOXP3+ cells in the thymic medulla, lymph nodes and spleen as compared to controls. **g** Relative mRNA expression level of *Aire* determined by RT-qPCR analysis of thymus tissue from control and *Spink5* cKO littermate mice at the age of 4-5 weeks. The transcription factor AIRE regulates the expression of tissue-restricted antigens in medullary thymic epithelial cells including keratinocyte-specific autoantigens such as LEKTI. Loss-of-function mutations in AIRE cause autoimmune diseases in humans and mice. The mRNA level of *Aire* in *Spink5* cKO thymi was decreased slightly, but not significantly. **h** Analysis of the TCRβ repertoire of thymocytes from control and *Spink5* cKO littermates by 5' RACE RT-PCR followed by deep sequencing of Trav-Traj junctions. Plot of principal component analysis/unsupervised hierarchical clustering analysis of Trav and Traj sequence data performed using PROMIDISα assay. The cluster of control and *Spink5* cKO samples is highlighted in green, while the positive sample cluster Xlf/XLF is marked in cyan. The depletion of DP thymocytes and the concomitant increase in the number of SP CD4+ and SP CD8+ cells in *Spink5* cKO thymi suggest rapid transition of thymocytes through the DP stage of development, when rearrangement of the T cell receptor α (TCRα) chain is initiated. Accelerated positive selection might result in incomplete V(D)J recombination of TCRα chains and thus lead to defective TCRα in SP thymocytes, affecting TCR repertoire and eventually contributing to autoimmune disease. However, TCRα repertoire analyses of *Spink5* cKO thymocytes did not reveal any differences from control mice. **i-j** Relative mRNA expression level of *Sphk1* (**i**) and *Cd69* (**j**) – two molecules involved in the regulation of T cell emigration – determined by RT-qPCR analysis of thymus tissue from control and *Spink5* cKO littermate mice at the age of 4-5 weeks. The accumulation of SP thymocytes in *Spink5* cKO thymi could reflect impaired emigration of mature T cells from the thymus. Increased expression of Sphingosine kinase 1 (Sphk1) in the thymus during sepsis leads to high levels of sphingosine-1 phosphate (S1P) in the thymus, which disturbs the S1P gradient and thus blocks egress of mature thymocytes. Increased levels of CD69 are also known to block emigration of mature thymocytes from the thymus. There were no significant changes of *Sphk1* and *Cd69* mRNA expression levels in thymus of *Spink5* cKO mice as compared to control littermates, arguing against a defect in T cell emigration in *Spink5* cKO thymi. **k-p** Relative mRNA expression levels of the cytokines *Tnf* (**k**), *Il6* (**l**), *Ifng* (**m**), *Cxcl1* (**n**), *Il17a* (**o**), and *Il22* (**p**) determined by RT-qPCR analysis of thymus tissue from control and *Spink5* cKO littermate mice at the age of 4-5 weeks. Plots in (**a**, **b**), (**f-g**) and (**i-p**) show means (bars) and scatter plots, where dots correspond to values measured for individual mice (n≥5 per group). Statistical significance was determined using a two-tailed non-parametric Wilcoxon matched-pairs signed rank test (**a**, **b**) or two-tailed unpaired non-parametric Mann-Whitney test (**f-g**, **i-p**): $^*p<0.05$, $^{***}p<0.001$, ns (not significant). Control mice are *Spink5*$^{fl/fl}$ and/or *Spink5*$^{fl/-}$; *Spink5* cKO mice are *KRT14-CreERT2*$^{(Tg/0)}$/*Spink5*$^{fl/fl}$ and/or *KRT14-CreERT2*$^{(Tg/0)}$/*Spink5*$^{fl/-}$.

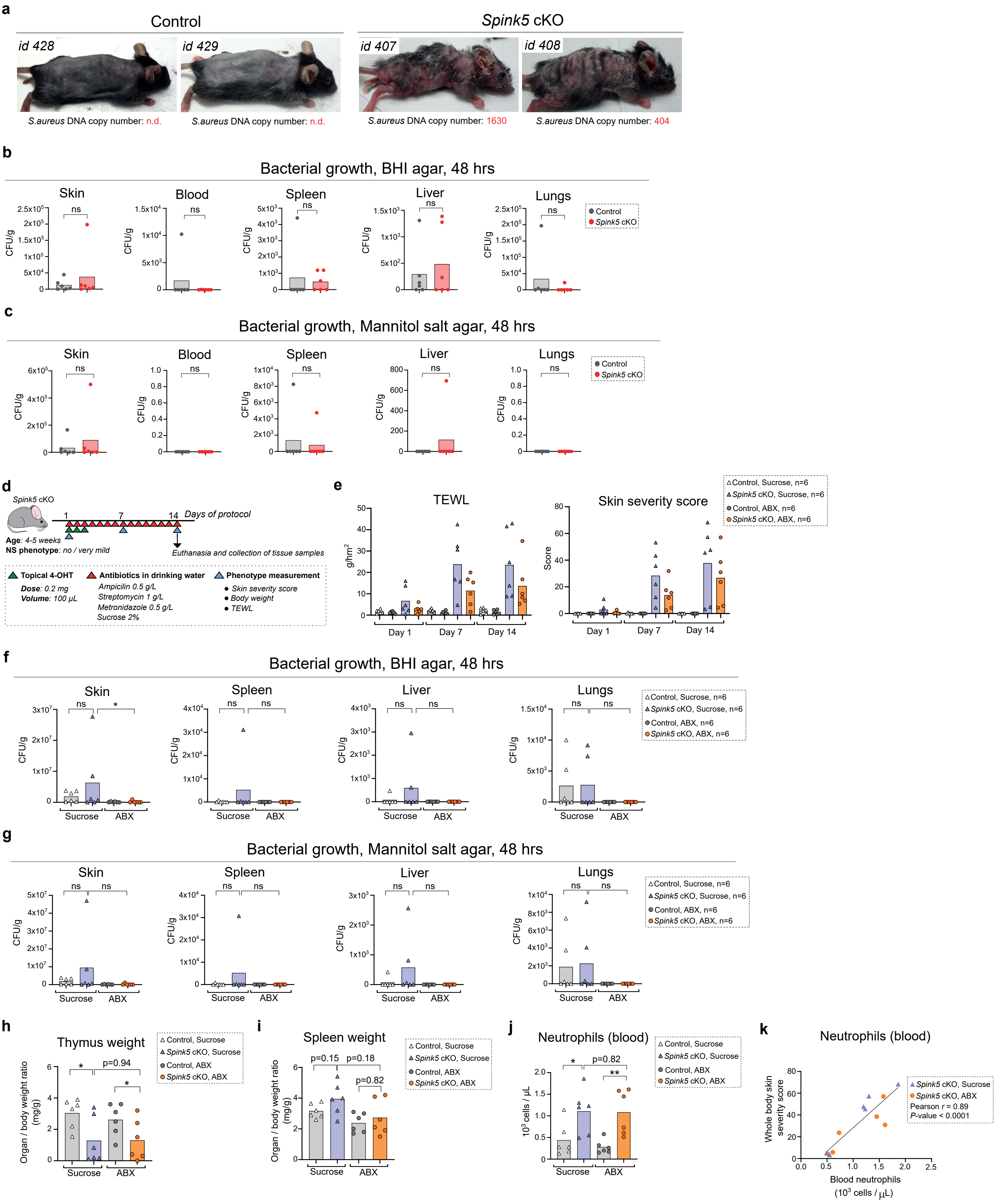

Supplementary Fig. 18: Role of bacteria in skin and systemic inflammation of *Spink5* cKO mice.
a Detection of *Staphylococcus aureus* DNA in skin swab samples from control (left panel) and *Spink5* cKO mice (right panel). Back skin was shaved one day before collection of skin swab samples from the back skin of each mouse. The collection of skin swab samples was done on the same day for all mice. *Spink5* cKO mice were housed in the same cage, while the control mice were housed together in a different cage. The *Spink5* cKO mice have severe (id407) and moderate (id408) skin lesions due to tamoxifen-independent, spontaneous CreERT2 activation. Analyses of skin swab samples by quantitative PCR for the presence of *Staphylococcus aureus* were performed at Charles River laboratories. The estimated DNA copy number obtained by *S.aureus* qPCR assay is indicated in red for each mouse below the corresponding image. n.d. = not detected. **b** Quantification of bacterial load in skin, blood,

spleen, liver and lungs of control and *Spink5* cKO mice by culture of tissue homogenates on Brain and Heart Infusion (BHI) agar plates. Colony forming units (CFU) were counted 48 hours after incubation at 37°C in aerobic conditions. **c** Quantification of *S. aureus* bacterial load in skin, blood, spleen, liver and lungs of control and *Spink5* cKO mice by culture of tissue homogenates on mannitol salt agar plates. Colony forming units (CFU) were counted 48 hours after incubation at 37°C in aerobic conditions. **d** Schematic of protocol for treatment of control and *Spink5* cKO mice with broad-spectrum antibiotics (ABX) cocktail. Mice in the placebo group received 2% sucrose in the drinking water. **e** Plots of transepidermal water loss (TEWL) values and whole-body skin severity scores measured in control and *Spink5* cKO mice during the treatment protocol described in (**d**). **f** Quantification of bacterial load in skin, spleen, liver and lungs of antibiotics (ABX)- or sucrose-treated control and *Spink5* cKO mice by culture of tissue homogenates on BHI agar plates. Colony forming units (CFU) were counted 48 hours after incubation at 37°C in aerobic conditions. **g** Quantification of bacterial load in skin, spleen, liver and lungs of antibiotics (ABX)- or sucrose-treated control and *Spink5* cKO mice by culture of tissue homogenates on mannitol salt agar plates. Colony forming units (CFU) were counted 48 hours after incubation at 37°C in aerobic conditions. **h**-**i** Plot of thymus weight/body weight ratios (**h**) and spleen weight/body weight ratios (**i**) (organ weight in milligrams/body weight in grams) in sucrose- and ABX-treated control and *Spink5* cKO mice. **j** Neutrophil counts measured in peripheral blood from sucrose- and ABX-treated control and *Spink5* cKO mice at the end of the treatment protocol. **k** Scatter plot of correlation between blood neutrophil counts and whole-body skin severity score of sucrose- and ABX-treated *Spink5* cKO mice. Regression line, Pearson correlation coefficient *r* and *P*-value are indicated. Plots in (**b**-**c**) and (**f**-**j**) show means (bars) and scatter plots, where dots correspond to values measured for individual mice (n=6 mice per group). Statistical significance was determined using two-tailed unpaired non-parametric Mann-Whitney test: *p<0.05, ns (not significant). Control mice are $Spink5^{fl/fl}$ and/or $Spink5^{fl/-}$; *Spink5* cKO mice are $KRT14\text{-}CreERT2^{(Tg/0)}/Spink5^{fl/fl}$ and/or $KRT14\text{-}CreERT2^{(Tg/0)}/Spink5^{fl/-}$.

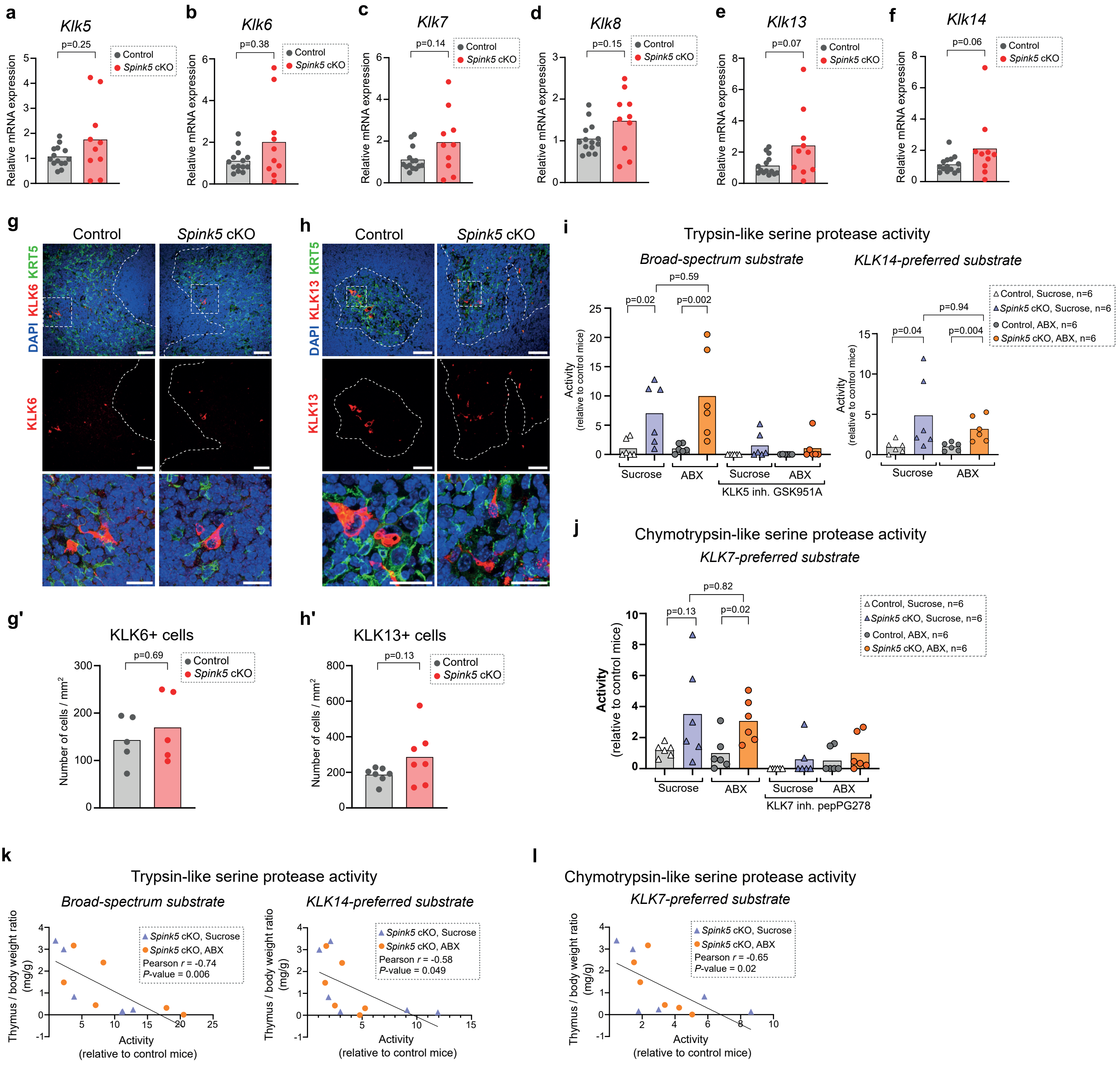

Supplementary Fig. 19: Expression and activity of kallikrein-related peptidases in thymus of *Spink5* cKO mice.

a-f Relative mRNA expression level of *Klk5* (**a**), *Klk6* (**b**), *Klk7* (**c**), *Klk8* (**d**), *Klk13* (**e**) and *Klk14* (**f**) in thymus of control and *Spink5* cKO mice determined by RT-qPCR. **g-h** Double immunofluorescence staining of (**g**) KLK6 (red) and the marker of medullary thymic epithelial cells Keratin 5 (KRT5, green) and (**h**) KLK13 and KRT5 in paraffin sections of thymus from control and *Spink5* cKO mice. Thymic medulla is outlined by a white dashed line. The region marked with white dashed-line rectangles in the upper panels is the magnified image shown in the lowermost panels. Scale bars: 50 µm, magnified image: 25 µm. **g'-h'** Number of KLK6+ cells (**g'**) and KLK13+ cells (**h'**) quantified in immunofluorescence staining images of the thymic medulla region in control and *Spink5* cKO mice. Images are representative of immunofluorescence staining performed on thymus samples of at least 4 different mice per group. **i** Measurements of trypsin-like serine protease activity in protein extracts from thymus of sucrose- and antibiotics (ABX)-treated control and *Spink5* cKO mice using the broad-spectrum fluorogenic substrate for trypsin-like serine proteases Boc-VPR-amc (left panel) and the KLK14-preferred fluorogenic peptide substrate Ac-WAVR-amc (right panel). **j** Measurements of chymotrypsin-like serine protease activity in protein extracts from thymus of sucrose- and ABX-treated control and *Spink5* cKO mice using the KLK7-preferred fluorogenic peptide substrate KHLY-amc. **k-l** Scatter plot of correlation between trypsin-like serine protease activity (**k**) or chymotrypsin-like serine protease activity (**l**) and thymus/body weight ratio (thymus weight in milligrams/body weight in grams) measured in sucrose- and ABX-treated *Spink5* cKO mice.

In (**i**) and (**j**), activity is expressed as a ratio of the fluorescence intensity value measured in each thymus sample to the mean of the fluorescence intensity values measured in thymus extracts from control mice. The addition of the KLK5-specific inhibitor GSK951A or the KLK7-specific inhibitor pepPG278 serves as control to estimate the percent of trypsin-like or chymotrypsin-like serine protease activities due to KLK5 or KLK7 activation, respectively. Data in (**a**-**f**), (**g'**-**h'**) and (**i**-**j**) show means (bars) and scatter plots, where dots correspond to values measured for individual mice (n≥4 per group). Statistical significance was determined using two-tailed unpaired non-parametric Mann-Whitney test and *P*-values are indicated on each plot. Control mice are *Spink5*$^{fl/fl}$ and/or *Spink5*$^{fl/-}$; *Spink5* cKO mice are *KRT14-CreERT2*$^{(Tg/0)}$/*Spink5*$^{fl/fl}$ and/or *KRT14-CreERT2*$^{(Tg/0)}$/*Spink5*$^{fl/-}$.

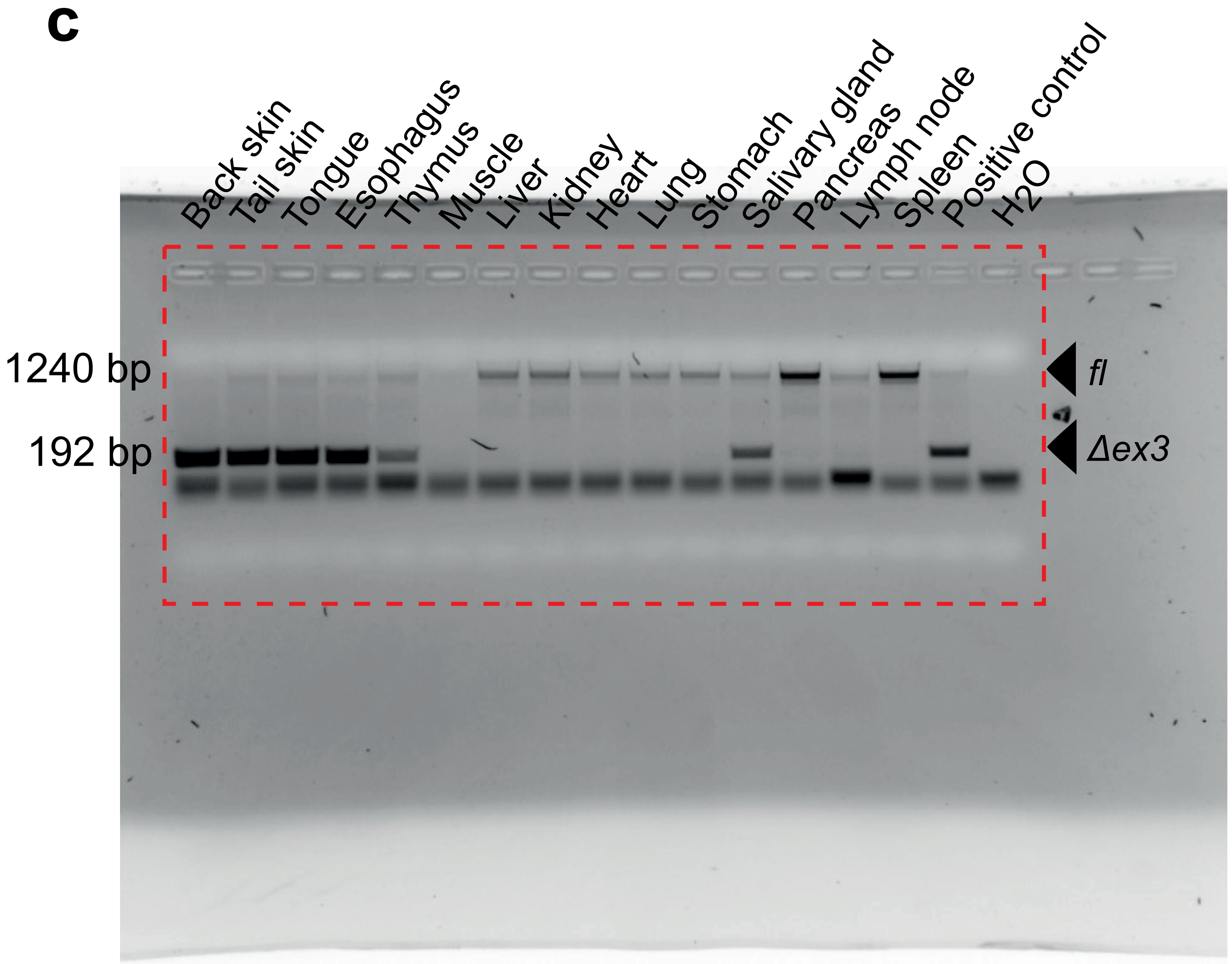

Supplementary Fig. 20: Uncropped and unedited gel image for Fig. 1c.

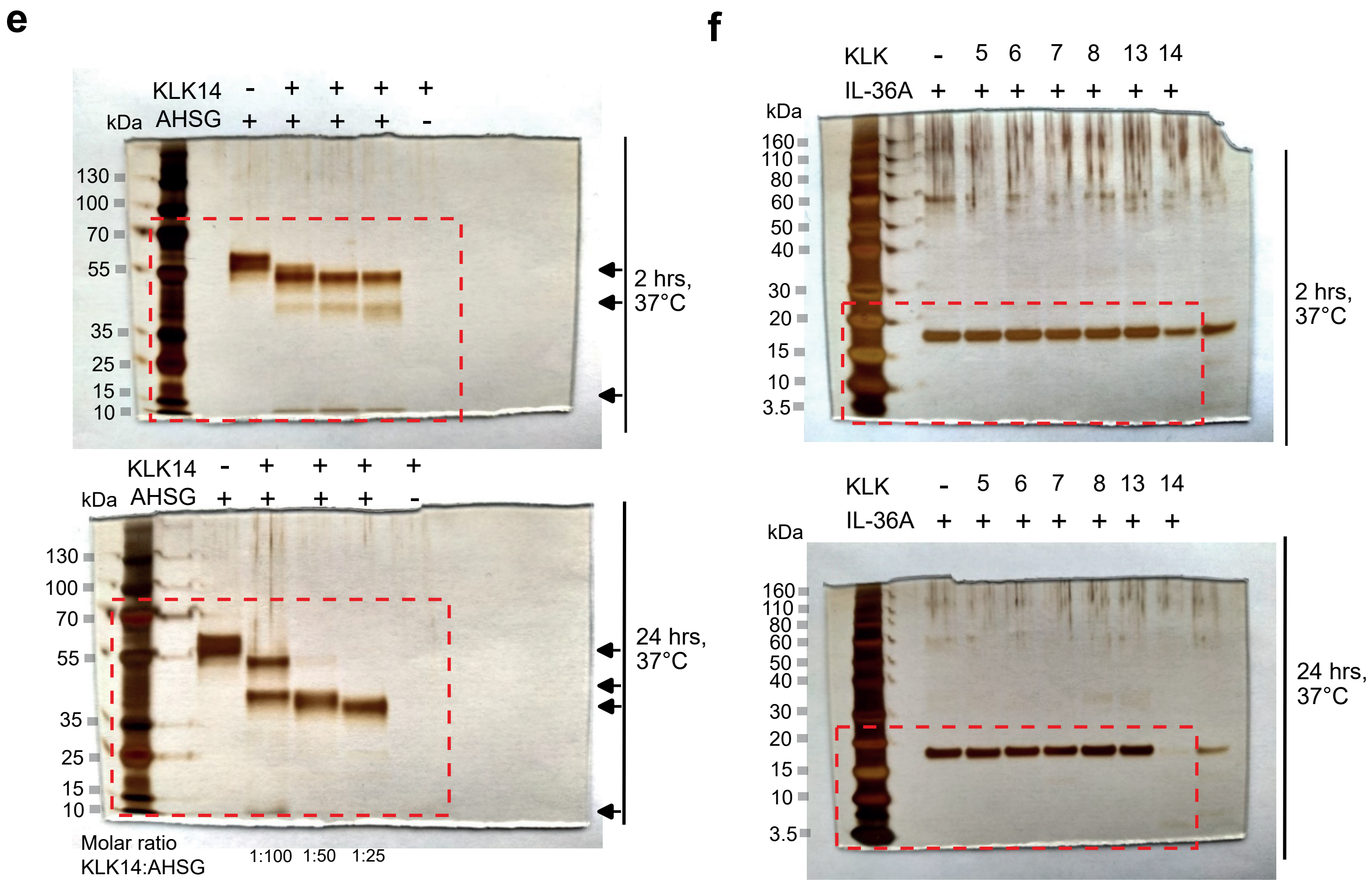

Supplementary Fig. 21: Uncropped and unedited gel images for Fig. 5e and Fig. 5f.

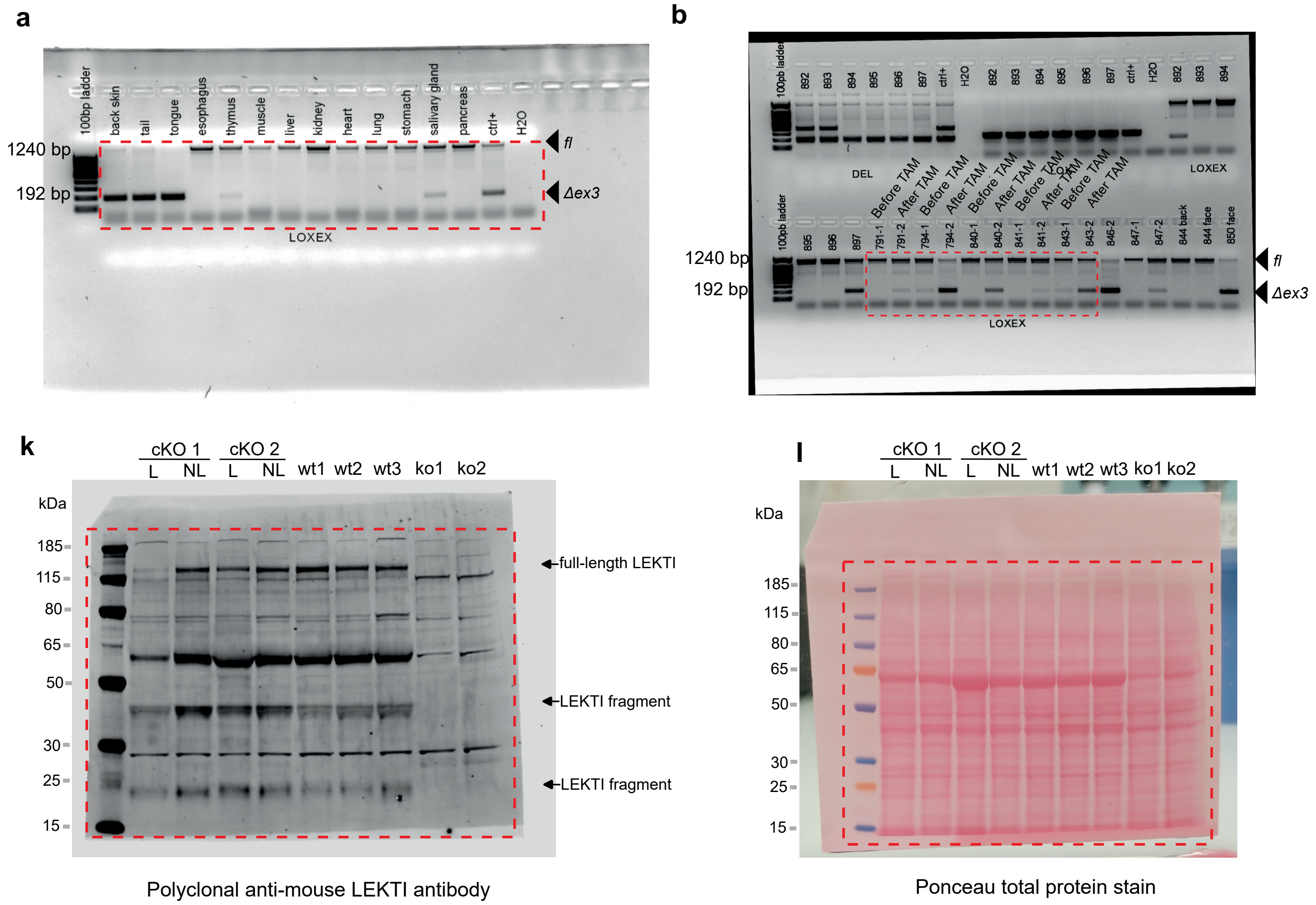

Supplementary Fig. 22: Uncropped and unedited gel/blot images for Supplementary Fig. 2a-b and Supplementary Fig. 2k-l.

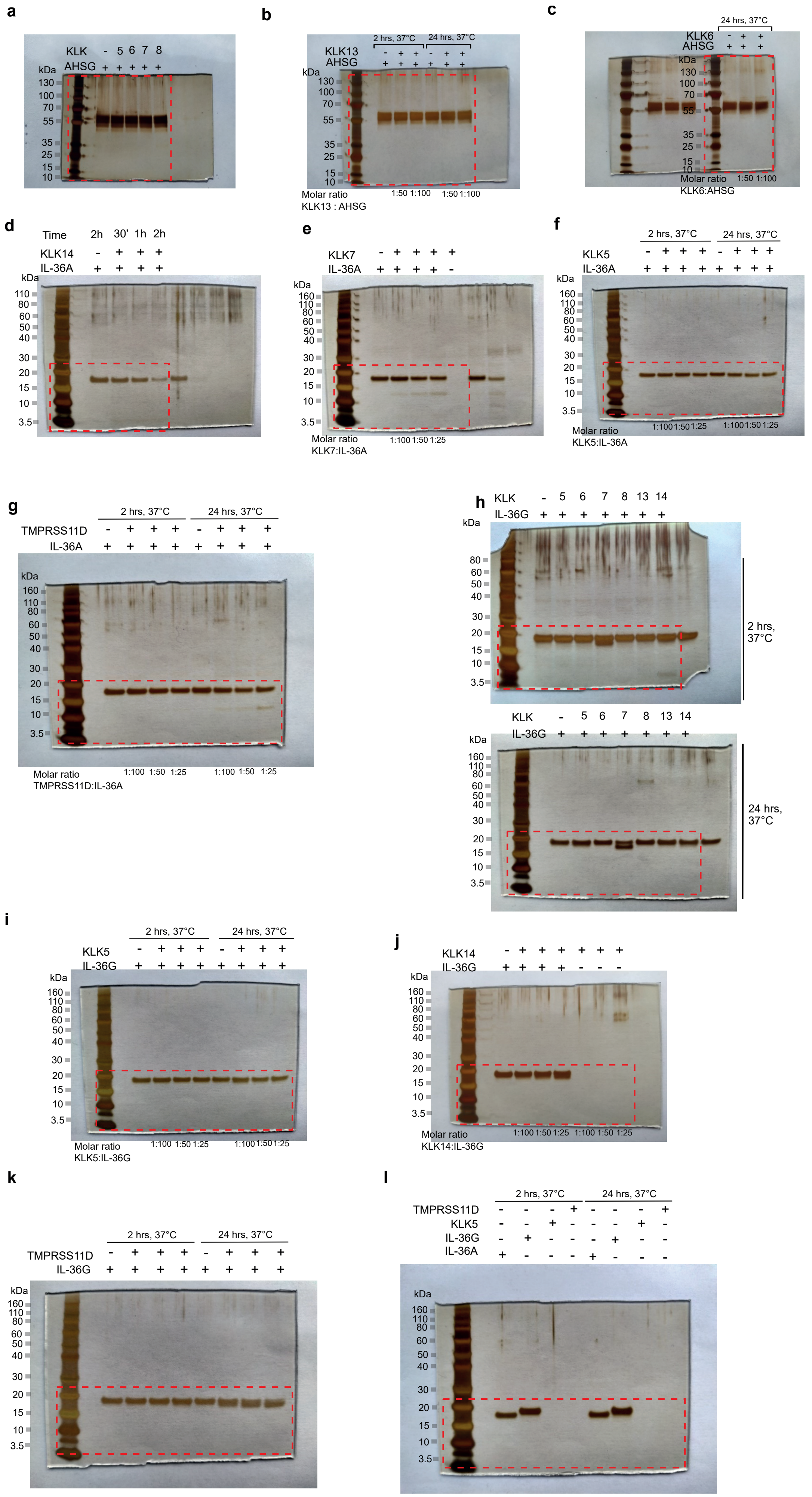

Supplementary Fig. 23: Uncropped and unedited gel images for Supplementary Fig. 11.
