# Supplementary material for: Comparative analyses of Netherton syndrome patients and Spink5 conditional knock-out mice uncover disease-relevant pathways
Source: Commun Biol. 2024 Feb 5;7:152. doi: 10.1038/s42003-024-05780-y (PMC10844249; doi:10.1038/s42003-024-05780-y)
Supplement: Supplementary file 3 — Description of Additional Supplementary Files [file 42003_2024_5780_MOESM3_ESM.pdf]

Description of Additional Supplementary Files

File name: Supplementary Data 1
Description: GO term enrichment analysis of the differentially up-regulated and down-regulated genes shared between lesional skin of *Spink5* cKO mice and NS patients. List of the significantly enriched biological process GO terms identified in the set of differentially expressed genes shared between lesional skin of *Spink5* cKO mice and lesional skin of NS patients. The differentially expressed genes annotated to each enriched GO term are listed with the corresponding adjusted *P*-value and expression fold change values (log2) for human genes (columns marked in blue) and their mouse homologues (columns marked in yellow). The GO terms are grouped in 8 categories (blue text) according to similarity of biological process.

File name: Supplementary Data 2
Description: Comparison of gene expression between skin of NS mouse models and lesional skin of NS, psoriasis and atopic dermatitis patients. The group of genes annotated to the different categories of significantly enriched GOs in the skin transcriptome of *Spink5* cKO, Tg.*hKLK5* or *Spink5*$^{-/-}$ mice were each compared to the corresponding group of genes in the skin transcriptome of NS, psoriasis and atopic dermatitis patients using Pearson correlation analysis. Fisher's r-to-z test was used to compare the Pearson correlation coefficients in each comparison. Each column contains the resulting *P*-values.

File name: Supplementary Data 3
Description: GO term enrichment analysis of the differentially up-regulated and down-regulated proteins shared between lesional skin of *Spink5* cKO mice and NS patients. List of the significantly enriched biological process GO terms identified in the set of differentially expressed proteins shared between lesional skin of *Spink5* cKO mice and lesional skin of NS patients. The differentially expressed proteins annotated to each enriched GO term are listed with the corresponding adjusted *P*-value and expression fold change values (log2) for human proteins (columns marked in blue) and their mouse homologues (columns marked in yellow). The GO terms are grouped into 8 categories (blue text) according to the similarity of biological processes.

File name: Supplementary Data 4
Description: GO term enrichment analysis of the differentially up-regulated and down-regulated genes in lymph nodes of *Spink5* cKO mice. List of the significantly enriched biological process GO terms identified in the set of differentially expressed genes in lymph node of *Spink5* cKO mice. The differentially expressed genes annotated to each enriched GO term are listed with the corresponding adjusted *P*-value and expression fold change values (log2).

File name: Supplementary Data 5
Description: GO term enrichment analysis of the differentially up-regulated and down-regulated genes shared between lesional skin and lymph nodes of *Spink5* cKO mice. List of the significantly enriched biological process GO terms identified in the set of differentially expressed genes shared between lesional skin and lymph node in *Spink5* cKO mice. The

differentially expressed genes annotated to each enriched GO term are listed with the corresponding adjusted *P*-value and expression fold change values (log2).

File name: Supplementary Data 6
Description: The source data behind the graphs in the paper.
