# Supplementary material for: Comparative analyses of Netherton syndrome patients and Spink5 conditional knock-out mice uncover disease-relevant pathways
Source: Commun Biol. 2024 Feb 5;7:152. doi: 10.1038/s42003-024-05780-y (PMC10844249; doi:10.1038/s42003-024-05780-y)
Supplement: Supplementary file 10 — Reporting Summary [file 42003_2024_5780_MOESM10_ESM.pdf]

nature portfolio

Corresponding author(s): Evgeniya Petrova and Alain Hovnanian

Last updated by author(s): Jan 3, 2024

Reporting Summary

Nature Portfolio wishes to improve the reproducibility of the work that we publish. This form provides structure for consistency and transparency in reporting. For further information on Nature Portfolio policies, see our Editorial Policies and the Editorial Policy Checklist.

Statistics

For all statistical analyses, confirm that the following items are present in the figure legend, table legend, main text, or Methods section.

n/a	Confirmed	
☐	☒	The exact sample size (*n*) for each experimental group/condition, given as a discrete number and unit of measurement
☐	☒	A statement on whether measurements were taken from distinct samples or whether the same sample was measured repeatedly
☐	☒	The statistical test(s) used AND whether they are one- or two-sided<br>*Only common tests should be described solely by name; describe more complex techniques in the Methods section.*
☐	☒	A description of all covariates tested
☐	☒	A description of any assumptions or corrections, such as tests of normality and adjustment for multiple comparisons
☐	☒	A full description of the statistical parameters including central tendency (e.g. means) or other basic estimates (e.g. regression coefficient) AND variation (e.g. standard deviation) or associated estimates of uncertainty (e.g. confidence intervals)
☐	☒	For null hypothesis testing, the test statistic (e.g. *F*, *t*, *r*) with confidence intervals, effect sizes, degrees of freedom and *P* value noted<br>*Give P values as exact values whenever suitable.*
☒	☐	For Bayesian analysis, information on the choice of priors and Markov chain Monte Carlo settings
☒	☐	For hierarchical and complex designs, identification of the appropriate level for tests and full reporting of outcomes
☐	☒	Estimates of effect sizes (e.g. Cohen's *d*, Pearson's *r*), indicating how they were calculated

Our web collection on statistics for biologists contains articles on many of the points above.

Software and code

Policy information about availability of computer code

Data collection	Wallac EnVision Manager 1.12, LAS X (Leica Microsystems), NDP.scan (Hamamatsu), Nova Seq 6000 System, HiSeq 2000 System, BD FACS Diva v8, CFX Maestro Software (BioRad), Image Studio (Li-COR Biosciences), ProCyte Dx analyzer (Idexx).
Data analysis	Microsoft Excel, Fiji 2.11.0, FlowJo v10 (BD Biosciences), GraphPadPrism 9, NDP.view2, Python, RStudio, MaxQuant v1.6.14.0., QuPath 0.4.3, Image Studio (Li-COR Biosciences)

For manuscripts utilizing custom algorithms or software that are central to the research but not yet described in published literature, software must be made available to editors and reviewers. We strongly encourage code deposition in a community repository (e.g. GitHub). See the Nature Portfolio guidelines for submitting code & software for further information.

Data

Policy information about availability of data

All manuscripts must include a data availability statement. This statement should provide the following information, where applicable:

- Accession codes, unique identifiers, or web links for publicly available datasets
- A description of any restrictions on data availability
- For clinical datasets or third party data, please ensure that the statement adheres to our policy

The transcriptomic data were deposited at the Gene Expression Omnibus (GEO) data repository under accession number GSE224280 (Spink5 cKO skin transcriptome), GSE224409 (Spink5 cKO lymph node transcriptome), GSE224673 (Tg.hKLK5 skin transcriptome), and GSE224674 (Spink5-/- skin transcriptome).

Mass spectrometry data (mass spectrometry raw spectral files and intermediary identification search results) were uploaded to the MassIVE repository (part of the ProteomeXchange consortium). Data can be accessed using the MassIVE accession number MSV000091184 or the ProteomeXchange accession number PXD039796.

Human research participants

Policy information about studies involving human research participants and Sex and Gender in Research.

Reporting on sex and gender	*Use the terms sex (biological attribute) and gender (shaped by social and cultural circumstances) carefully in order to avoid confusing both terms. Indicate if findings apply to only one sex or gender; describe whether sex and gender were considered in study design whether sex and/or gender was determined based on self-reporting or assigned and methods used. Provide in the source data disaggregated sex and gender data where this information has been collected, and consent has been obtained for sharing of individual-level data; provide overall numbers in this Reporting Summary. Please state if this information has not been collected. Report sex- and gender-based analyses where performed, justify reasons for lack of sex- and gender-based analysis.*
Population characteristics	*Describe the covariate-relevant population characteristics of the human research participants (e.g. age, genotypic information, past and current diagnosis and treatment categories). If you filled out the behavioural & social sciences study design questions and have nothing to add here, write "See above."*
Recruitment	*Describe how participants were recruited. Outline any potential self-selection bias or other biases that may be present and how these are likely to impact results.*
Ethics oversight	*Identify the organization(s) that approved the study protocol.*

Note that full information on the approval of the study protocol must also be provided in the manuscript.

Field-specific reporting

Please select the one below that is the best fit for your research. If you are not sure, read the appropriate sections before making your selection.

☒ Life sciences ☐ Behavioural & social sciences ☐ Ecological, evolutionary & environmental sciences

For a reference copy of the document with all sections, see nature.com/documents/nr-reporting-summary-flat.pdf

Life sciences study design

All studies must disclose on these points even when the disclosure is negative.

Sample size	Sample size was determined according to our previous mouse studies as well as according to preliminary studies with Spink5 conditional knock-out mice analyzing the variability of their skin disease phenotype. Three to ten mice per group were estimated sufficient to detect differences between groups in most of the experiments involving skin tissue molecular analyses or transcriptomic analyses of lymph nodes. For flow cytometry analyses, a higher number of mice per group was used that allowed to correlate lymphoid organ phenotype with skin disease severity. Similarly, survival analyses and phenotype analyses of live mice (skin disease score, transepidermal water loss measurements) were performed with higher number of Spink5 conditional knock-out mice and control littermates due to variation of skin disease phenotype with time or between mice with different levels of spontaneous, tamoxifen-independent CreERT2 activation.
Data exclusions	No data were excluded from the analyses.
Replication	In vitro digestion experiments were repeated twice with similar results. The rest of the experiments were performed on at least five different mice per experimental group, unless otherwise indicated in the figure legends or Materials and Methods section.
Randomization	Littermate, sex-matched mice were randomly assigned to each group. In some experiments, Spink5 conditional knock-out mice and control (wildtype) mice were not littermates (age-matched) nor sex-matched, but they were assigned based on similarity of age (young adult mice between 3 and 8 weeks of age), so that the mean age of the mice in each experimental group is similar.
Blinding	In experiments involving phenotype measurements of live mice or histology / immunofluorescence microscopy of skin samples, blinding was not possible because the skin disease phenotype was easily visible and thus mice from transgenic/knock-out groups could be easily distinguished from control (wildtype) mice. In the experiment, where Wt control mice and Spink5 cKO mice were treated with antibiotics cocktail or sucrose only (placebo) in drinking water, there was no blinding.

Reporting for specific materials, systems and methods

We require information from authors about some types of materials, experimental systems and methods used in many studies. Here, indicate whether each material, system or method listed is relevant to your study. If you are not sure if a list item applies to your research, read the appropriate section before selecting a response.

Materials & experimental systems

n/a	Involved in the study
☐	☒ Antibodies
☒	☐ Eukaryotic cell lines
☒	☐ Palaeontology and archaeology
☐	☒ Animals and other organisms
☒	☐ Clinical data
☒	☐ Dual use research of concern

Methods

n/a	Involved in the study
☒	☐ ChIP-seq
☐	☒ Flow cytometry
☒	☐ MRI-based neuroimaging

Antibodies

Antibodies used

Antibodies used for flow cytometry:
CD3-FITC (BD Biosciences, #555274, Clone:17A2)
CD4-APC/Cy7 (BD Pharmingen, #552051, Clone: GK1.5)
CD8a-PE (Sony Biotechnology, #1103540, Clone: 53-6.7)
CD44-Pacific Blue (Biolegend, #103020, Clone: IM7)
CD25-PerCP/Cy5.5 (Sony Biotechnology, #1109560, Clone: 3C7)
TCRb-PE/Cy7 (Biolegend, #109222, Clone: H57-597)
TCR Vδ4-BV605 (BD Biosciences, #745116, Clone: GL2)
IgM-APC (Sony Biotechnology, #2632545, Clone: RMM-1)
CD45R/B220-PerpCP/Cy5.5 (Sony Biotechnology, #1116180, Clone: RA3-6B2)

Primary antibodies used for immunofluorescence staining of antigens on paraffin sections:
LEKTI (Cloud Clone Corp., #PAH145Mu01, polyclonal)
S100A8 (Cell Signaling Technology, #47310, Clone: E4F8V)
S100A9 (Cell Signaling Technology, #73425, Clone: D3U8M)
KRT6A (Biolegend, #905701, polyclonal)
LOR (Biolegend, #PRB-145P, polyclonal)
TSLP (R&D Systems, #AF555, polyclonal)
IL-36A (R&D Systems, #AF2297, polyclonal)
IL-24 (R&D Systems, #MAB2786, Clone: 303308)
IVL (Biolegend, #924401, polyclonal)
CDH1 (Cell Signaling Technology, #3195, Clone: 24E10)
KRT10 (Biolegend, #905401, polyclonal)
pSTAT3(Tyr705) (Cell Signaling Tecnology, #9145, Clone: D3A7)
FOXP3 (Cell Signaling Technology, #12653, Clone: D6O8R)
IL-17A (Abcam, #ab79056, polyclonal)
CD3 (Abcam, #ab11089, Clone: CD3-12)
KRT14 (Abcam, #ab181595, Clone: EPR17350)
Ki67 (Abcam, #ab15580, polyclonal)
CD19 (Cell Signaling Technology, #90176, Clone: D4V4B)
KRT10 (Biolegend, #905403, polyclonal)
KRT14 (Biolegend, #906004, polyclonal)
KRT5 (Biolegend, #905903, polyclonal)
CD34 (Abcam, # ab8158, monoclonal)
VIMENTIN (Cell Signaling Technology, #5741, monoclonal)
KLK5 (Abbexa, #abx100977, polyclonal)
KLK6 (Abbexa, # abx100979, polyclonal)
KLK7 (R&D Systems, # AF2624, polyclonal)
KLK13 (Abbexa, # abx129912, polyclonal)
KLK14 (Abcam, # ab278500, monoclonal)

Primary antibodies used for immunofluorescence staining of antigens on cryosections:

CD4 (BD Pharmingen, #553727, Clone: GK1.5)
CD3e (BD Pharmingen, #555273, Clone: 17A2)
CD8a (BD Pharmingen, #553027, Clone: 53-6.7)
CXCL3 (ThermoFisherScientific, #PA5-103136, polyclonal)
F4/80 (Abcam, #ab6640, Clone: CI:A3-1)
Ly-6G/C (Abcam, #ab2557, Clone: NIMP-R14)
DSP (Proteintech, #25318-1-AP, polyclonal)
FLG (Biolegend, #PRB-417)
DSG1 (Santa Cruz Biotechnology, #sc20114, polyclonal)

Primary antibodies used for Western blot:
LEKTI (Cloud Clone Corp., #PAH145Mu01, polyclonal)

Secondary antibodies used for immunofluorescence staining of tissue sections:
goat anti-rat AlexaFluor555 (ThermoFisherScientific, #A21434)
goat anti-rabbit AlexaFluor555 (ThermoFisherScientific, #A21430)

donkey anti-goat AlexaFluor555 (ThermoFisherScientific, #A11056)
goat anti-rat AlexaFluor488 (ThermoFisherScientific, #A11006)
donkey anti-chicken AlexaFluor488 (ThermoFischerScientific, #A78948)
goat anti-chicken AlexaFluor488 (ThermoFischerScientific, # A11039)

Secondary antibodies used for Western blot:
goat anti-rabbit IRDye 680RD (Li-COR Biosciences, # 926-68071)

Validation

Antibodies used for flow cytometry:
CD3-FITC (BD Biosciences, #555274, Clone:17A2)
BALB/c splenocytes were simultaneously stained with FITC-conjugated 17A2 (Cat. No. 555274) and PE-conjugated anti-mouse CD19 mAb 1D3 (Cat. No. 557399, left panel). BALB/c thymocytes were also stained with FITC-conjugated 17A2 mAb (Cat. No. 555274, right panel). Flow cytometry was performed on a BD FACScan™ flow cytometry system. 555274.pdf (bdbiosciences.com)

CD4-APC/Cy7 (BD Pharmingen, #552051, Clone: GK1.5)
BALB/c splenocytes were simultaneously stained with FITC-conjugated 1445-2C11 mAb (anti-mouse CD3e, Cat. No. 553061, both panels), and APC-Cy7 conjugated rat anti-mouse CD4 clone GK1.5 mAb (right panel), in the presence of BD Mouse Fc Block™ purified anti-mouse CD16/CD32 mAb 2.4G2 (Cat. No. 553141). Flow cytometry was performed on a BD FACSVantage™ SE Flow Cytometry System.
APC-Cy™7 Rat Anti-Mouse CD4 — 552051 (bdbiosciences.com)

CD8a-PE (Sony Biotechnology, #1103540, Clone: 53-6.7)
C57BL/6 mouse splenocytes were stained with CD8 (clone 53-6.7) PE (filled histogram) or rat IgG2a, κ PE isotype control (open histogram). Product Data Sheet (d177r6bz0xmhgl.cloudfront.net)

CD44-Pacific Blue (Biolegend, #103020, Clone: IM7)
Pacific Blue anti-mouse/human CD44 Antibody anti-CD44 - IM7 (biolegend.com)
C57BL/6 Mouse Splenocytes stained with IM7 Pacific Blue™

CD25-PerCP/Cy5.5 (Sony Biotechnology, #1109560, Clone: 3C7)
Con A-stimulated C57BL/7 splenocytes (Day 3) stained with 3C7 PerCP/Cy5.5
Product Data Sheet (d177r6bz0xmhgl.cloudfront.net)

TCRb-PE/Cy7 (Biolegend, #109222, Clone: H57-597)
C57BL/6 mouse splenocytes stained with H57-597 PE/Cyanine7
PE/Cyanine7 anti-mouse TCR beta chain Antibody anti-TCR beta chain - H57-597 (biolegend.com)

TCR Vδ4-BV605 (BD Biosciences, #745116, Clone: GL2)
Mouse (Tested in Development)
BV605 Hamster Anti-Mouse Vδ 4 TCR (bdbiosciences.com)

IgM-APC (Sony Biotechnology, #2632545, Clone: RMM-1)
C57BL/6 splenocytes stained with RMM-1 APC
Product Data Sheet (d177r6bz0xmhgl.cloudfront.net)

CD45R/B220-PerpCP/Cy5.5 (Sony Biotechnology, #1116180, Clone: RA3-6B2)
C57BL/6 mouse splenocytes stained with RA3-6B2 PerCP/Cy5.5 Product Data Sheet (d177r6bz0xmhgl.cloudfront.net)

Primary antibodies used for immunofluorescence staining of antigens on paraffin sections:
LEKTI (Cloud Clone Corp., #PAH145Mu01, polyclonal)
DAB staining on IHC-P; Samples: Mouse Esophagus Tissue; Primary Ab: 10μg/ml Rabbit Anti-Mouse SPINK
Polyclonal Antibody to Serine Peptidase Inhibitor Kazal Type 5 (SPINK5) | PAH145Mu01 | Mus musculus (Mouse) CLOUD-CLONE CORP.(CCC)

S100A8 (Cell Signaling Technology, #47310, Clone: E4F8V)
Immunohistochemical analysis of paraffin-embedded mouse spleen using S100A8 (E4F8V) XP® Rabbit mAb performed on the Leica® BOND™ Rx.
S100A8 (E4F8V) XP® Rabbit mAb | Cell Signaling Technology

S100A9 (Cell Signaling Technology, #73425, Clone: D3U8M)
Immunohistochemical analysis of paraffin-embedded mouse ovary using S100A9 (D3U8M) Rabbit mAb performed on the Leica® Bond™ Rx.
S100A9 (D3U8M) Rabbit mAb | Cell Signaling Technology

KRT6A (Biolegend, #905701, polyclonal)
Staining of Mouse Keratin 6, on mouse tongue at a 1:5000 dilution.
Purified anti-mouse Keratin 6A Antibody anti-Keratin 6A - Poly19057 (biolegend.com)

LOR (Biolegend, #PRB-145P, polyclonal)
IHC staining of purified anti-Loricrin antibody (clone Poly19051) on formalin-fixed paraffin-embedded human skin tissue.
Purified anti-Loricrin Antibody anti-Loricrin - Poly19051 (biolegend.com)

TSLP (R&D Systems, #AF555, polyclonal)
IHC analysis shows abolishment of Tslp expression in Spink5-/-Klk5-/- skin as compared to Spink5-/-; KLK5 Inactivation Reverses Cutaneous Hallmarks of Netherton Syndrome | PLOS Genetics
Detects mouse TSLP in direct ELISAs and Western blots. Mouse TSLP Antibody AF555: R&D Systems (rndsystems.com)

IL-36A (R&D Systems, #AF2297, polyclonal)
Detects mouse IL 36 alpha /IL 1F6 in direct ELISAs and Western blots.
Mouse IL-36 alpha /IL-1F6 Antibody AF2297: R&D Systems (rndsystems.com)
For IHC staining of IL-36α, paraffin sections were treated with 0.6% H2O2 (in PBS) to block the endogenous peroxidase activity before antigen retrieving with citric buffer (10 mmol/l citric acid, pH 6). Slides were then blocked with 5% normal rabbit serum (Vector Laboratories) and incubated with goat polyclonal anti–mouse IL-36α antibody (R&D Systems, AF2297, dilution 1:100).
Disrupting the IL-36 and IL-23/IL-17 loop underlies the efficacy of calcipotriol and corticosteroid therapy for psoriasis - PMC (nih.gov)

IL-24 (R&D Systems, #MAB2786, Clone: 303308)
Detects mouse IL-24 in direct ELISAs and Western blots.
Mouse IL-24 Antibody MAB2786: R&D Systems (rndsystems.com)
Test in IHC of mouse skin in this study. Specific staining of epidermis in Spink5 cKO mice was observed.

IVL (Biolegend, #924401, polyclonal)
IHC staining of anti-Involucrin antibody (clone Poly19244) on formalin-fixed paraffin-embedded mouse skin tissue.
Anti-Involucrin Antibody anti-Involucrin - Poly19244 (biolegend.com)

CDH1 (Cell Signaling Technology, #3195, Clone: 24E10)
Immunohistochemical analysis of paraffin-embedded human prostate adenocarcinoma using E-Cadherin (24E10) Rabbit mAb performed on the Leica BOND Rx.
E-Cadherin (24E10) Rabbit mAb | Cell Signaling Technology

KRT10 (Biolegend, #905401, polyclonal)
Keratin 10 Polyclonal Antibody, Purified (biolegend.com)

pSTAT3(Tyr705) (Cell Signaling Tecnology, #9145, Clone: D3A7)
Immunohistochemical analysis of paraffin-embedded Apc (min/+) mouse intestine, using Phosho-Stat3 (Tyr705) (D3A7) XP® Rabbit mAb.
Phospho-Stat3 (Tyr705) (D3A7) XP® Rabbit mAb | Cell Signaling Technology

FOXP3 (Cell Signaling Technology, #12653, Clone: D6O8R)
Immunohistochemical analysis of paraffin-embedded 4T1 metastatic tumor in mouse lung using FoxP3 (D6O8R) Rabbit mAb performed on the Leica® BOND™ Rx.
FoxP3 (D6O8R) Rabbit mAb | Cell Signaling Technology

IL-17A (Abcam, #ab79056, polyclonal)
Immunohistochemical analysis of paraffin-embedded Mouse Colon Tissue using ab79056 at 2 µg/mL.
Anti-IL-17A antibody (ab79056) | Abcam

CD3 (Abcam, #ab11089, Clone: CD3-12)
Immunohistochemical analysis of paraffin-embedded mouse spleen tissue labeling CD3 with ab11089 at 1/200 dilution, followed by ready to use Goat Anti-Rat IgG H&L (HRP polymer) (ab214882).
Anti-CD3 antibody [CD3-12] (ab11089) | Abcam

KRT14 (Abcam, #ab181595, Clone: EPR17350)
Immunohistochemical analysis of paraffin-embedded mouse skin tissue labeling Cytokeratin 14 with ab181595 at 1/2000 dilution, followed by prediluted HRP polymer for Rabbit/Mouse IgG.
Recombinant Anti-Cytokeratin 14 antibody [EPR17350] - Cytoskeleton Marker KO Tested (ab181595) (abcam.com)

Ki67 (Abcam, #ab15580, polyclonal)
IHC image of ab15580 staining in mouse spleen formalin fixed paraffin embedded tissue section, performed on a Leica BondTM system using the standard protocol B.
Anti-Ki67 antibody KO Tested (ab15580) | Abcam

CD19 (Cell Signaling Technology, #90176, Clone: D4V4B)
Immunohistochemical analysis of paraffin-embedded mouse spleen using CD19 (Intracellular Domain) (D4V4B) XP® Rabbit mAb.
CD19 Antibody (D4V4B) | Cell Signaling Technology

KRT10 (Biolegend, #905403, polyclonal)
Immunolabeling of mouse skin sections
Transcription factor E4F1 is essential for epidermal stem cell maintenance and skin homeostasis | PNAS
IHC staining of anti-Keratin 10 antibody (Poly19054) on formalin-fixed paraffin-embedded human skin tissue. Purified anti-Keratin 10 Antibody anti-Keratin 10 - Poly19054 (biolegend.com)

KRT14 (Biolegend, #906004, polyclonal)
IHC staining of purified anti-Keratin 14 antibody (Poly9060) on formalin-fixed paraffin-embedded canine skin tissue. Purified anti-Keratin 14 Antibody anti-Keratin 14 - Poly9060 (biolegend.com)

KRT5 (Biolegend, #905903, polyclonal)
IHC staining of anti-Keratin 5 antibody (Poly90590) on formalin-fixed paraffin-embedded human skin tissue. Purified anti-Keratin 5 Polyclonal Chicken Antibody anti-Keratin 5 - Poly9059 (biolegend.com)

CD34 (Abcam, # ab8158, monoclonal)
IHC image of CD34 staining in a formalin fixed paraffin embedded mouse lung tissue section, performed on a Leica Bond™ system using the standard protocol B. Anti-CD34 antibody [MEC 14.7] (ab8158) | Abcam

VIMENTIN (Cell Signaling Technology, #5741, monoclonal)
Immunohistochemical analysis of paraffin-embedded human squamous cell lung carcinoma using Vimentin (D21H3) XP® Rabbit mAb performed on the Leica® BOND™ Rx. Vimentin (D21H3) XP® Rabbit mAb | Cell Signaling Technology

KLK5 (Abbexa, #abx100977, polyclonal)
IHC-P analysis of Mouse stomach tissue, using KLK5 antibody (20 µg/ml) with DAB staining. Kallikrein 5 (KLK5) Antibody | Abbexa Ltd

KLK6 (Abbexa, # abx100979, polyclonal)
Immunofluorescence staining on paraffin sections of salivary glands from WT and Spink5 cKO mice.

KLK7 (R&D Systems, # AF2624, polyclonal)
Immunofluorescence staining on paraffin sections of skin from WT and Spink5 cKO mice.

KLK13 (Abbexa, # abx129912, polyclonal)
Immunofluorescence staining on paraffin sections of salivary glands from WT and Spink5 cKO mice.

KLK14 (Abcam, # ab278500, monoclonal)
Immunohistochemical analysis of paraffin-embedded Mouse esophagus tissue labelling Thyroid Peroxidase/TPO with ab278500 at 1/5000 (0.585 ug/ml) dilution followed by a ready to use LeicaDS9800 (Bond® Polymer Refine Detection) was used. Recombinant Anti-Kallikrein 14 antibody [EPR23570-293] (ab278500) | Abcam
Immunofluorescence staining on paraffin sections of skin from WT and Spink5 cKO mice.

Primary antibodies used for immunofluorescence staining of antigens on cryosections:

CD4 (BD Pharmingen, #553727, Clone: GK1.5)
This antibody is routinely tested by flow cytometric analysis. Other applications were tested at BD Biosciences Pharmingen during antibody development only or reported in the literature.
Search Results (bdbiosciences.com)

CD3e (BD Pharmingen, #555273, Clone: 17A2)
This antibody is routinely tested by flow cytometric analysis. Other applications were tested at BD Biosciences Pharmingen during antibody development only or reported in the literature.
Search Results (bdbiosciences.com)

CD8a (BD Pharmingen, #553027, Clone: 53-6.7)
Immunohistochemistry-frozen Tested During Development
Search Results (bdbiosciences.com)

CXCL3 (ThermoFisherScientific, #PA5-103136, polyclonal)
Immunohistochemistry analysis of paraffin-embedded CXCL3 in mouse kidney tissue. Antigen retrieval was performed using citrate buffer. Samples were blocked with blocking buffer (1.5 hr, 22°C), incubated with CXCL3 polyclonal antibody (Product # PA5-103136) using a dilution of 1:100 (1.5 hr, 22°C), followed by HRP conjugated goat anti-rabbit.
CXCL3 Polyclonal Antibody (PA5-103136) (thermofisher.com)

F4/80 (Abcam, #ab6640, Clone: CI:A3-1)
ab6640 staining F4/80 in mouse bone marrow cells by immunocytochemistry/ immunofluorescence.
Anti-F4/80 antibody [CI:A3-1] - Macrophage Marker (ab6640) | Abcam

Ly-6G/C (Abcam, #ab2557, Clone: NIMP-R14)
Frozen sections of mouse spleen. ab2557 was used in a concentration of 5 µg/ml.
Anti-Neutrophil antibody [NIMP-R14] (ab2557) | Abcam

DSP (Proteintech, #25318-1-AP, polyclonal)
Positive IHC detected in mouse heart tissue
Desmoplakin antibody (25318-1-AP) | Proteintech (ptglab.com)

FLG (Biolegend, #PRB-417)
Immunofluorescence staining of filaggrin protein in ear skin of WT and KO-D mice.
Frontiers | Essential Role of STAT3 Signaling in Hair Follicle Homeostasis (frontiersin.org)
Purified anti-Filaggrin Antibody anti-Filaggrin - Poly19058 (biolegend.com)

DSG1 (Santa Cruz Biotechnology, #sc20114, polyclonal)
Immunohistochemical analysis of desmoglein-1 (Dsg-1) expression in WT and Tg-KLK5 skin sections. Transgenic kallikrein 5 mice reproduce major cutaneous and systemic hallmarks of Netherton syndrome - PMC (nih.gov), Spink5-deficient mice mimic Netherton syndrome through degradation of desmoglein 1 by epidermal protease hyperactivity - PubMed (nih.gov)
dsg1 Antibody (B-11) | SCBT - Santa Cruz Biotechnology

Primary antibodies used for Western blot:
LEKTI (Cloud Clone Corp., #PAH145Mu01, polyclonal)
Western blot of recombinant mouse SPINK5. https://www.cloud-clone.com/products/PAH145Mu01.html
In the present study, the antibody was validated as specific for mouse SPINK5 by analyzing in parallel skin samples from Spink5-/- mice (full Spink5 knock-out mice).

Animals and other research organisms

Policy information about studies involving animals; ARRIVE guidelines recommended for reporting animal research, and Sex and Gender in Research

Laboratory animals	Genetically modified mice (species Mus musculus) were used in this study. All mice were of C57BL/6JRj strain. Skin samples from Spink5 constitutive knock-out mice were collected from newborn mice as soon as possible after birth. Skin samples were collected from 3-week-old transgenic hKLK5 mice and non-transgenic (wild-type) littermates. Spink5 conditional knock-out mice and littermate controls at the age of 4-12 weeks were used in this study.
Wild animals	No wild animals were used in this study.
Reporting on sex	Both male and female mice were used in this study. Information about the sex of the animals used in this study has been collected and is provided in the source data related to this study. Sex-based analyses were not performed, since both male and female transgenic/knock-out mice show similar skin disease phenotype.
Field-collected samples	No field-collected samples were used in this study.
Ethics oversight	Mouse colony maintenance and experimental procedures were performed in accordance with the ethical standards set by the national legislation and revised by the ethical committee for animal experimentation at Imagine Institute and approved by the French Ministry of Higher Education, Research and Innovation.

Note that full information on the approval of the study protocol must also be provided in the manuscript.

Flow Cytometry

Plots

Confirm that:

☒ The axis labels state the marker and fluorochrome used (e.g. CD4-FITC).

☒ The axis scales are clearly visible. Include numbers along axes only for bottom left plot of group (a 'group' is an analysis of identical markers).

☒ All plots are contour plots with outliers or pseudocolor plots.

☒ A numerical value for number of cells or percentage (with statistics) is provided.

Methodology

Sample preparation	Lymphoid organs were collected from 4-week-old Spink5 cKO mice (KRT14-CreERT2(Tg/0)/Spink5fl/fl and/or KRT14-CreERT2(Tg/0)/Spink5fl/-) and control littermate mice (Spink5fl/fl and/or Spink5fl/-). Single-cell suspensions from the whole thymus, spleen, and two inguinal lymph nodes per mouse were obtained by grinding the tissue with a syringe plunger against 70 µm mesh of a cell strainer in cold staining buffer (2 mM EDTA PBS buffer supplemented with 5% fetal bovine serum). Cell number and viability were determined using trypan blue dye and TC20 automated cell counter (Bio-Rad). Cells (1 million viable cells) were re-suspended in staining buffer containing a mix of fixable aqua dead cell stain (ThermoFisherScientifc, #L34957) and fluorescently-labelled antibodies for detection of B cells (anti-IgM, anti-B220) and T cells (anti-CD3, anti-CD4, anti-CD8) in lymph nodes and spleens, or thymocytes (anti-TCRbeta, anti-TCRdelta, anti-CD44, anti-CD25) and incubated for 30 minutes at 4°C. Cells were washed once in cold staining buffer and then fixed with medium A reagent (Fix&Perm kit, ThermoFisherScientific, #GAS001). Fixed cells were washed, re-suspended in staining buffer and analyzed using LSRFortessa X-20 flow cytometer (BD Biosciences).
Instrument	LSRFortessa X-20 flow cytometer (BD Biosciences)
Software	Collection of data was done using BD FACSDiva software. Analysis of data was done using FlowJo v10 (BD Biosciences).
Cell population abundance	Cell sorting was not performed in this study.
Gating strategy	Gating strategy for flow cytometry analyses of single cell suspensions from thymus: thymocytes (FCS-A vs SSC-A) were gated for single cells (SSC-A vs SSC-H). Single cells were further gated depending on cell viability. Cells expressing CD4 or CD8 were identified within the live cells gate (DCM-). Cells expressing CD44 and/or CD25 were identified within the CD4-CD8- double-negative (DN) cells gate. Gates are indicated with black or red contours and selection of events from each gate is indicated with a black or red arrow, respectively.<br>Gating strategy for flow cytometry analyses of single cell suspensions from inguinal lymph node and spleen: lymphocytes (FSC-A vs SSC-A) were gated for single cells (SSC-A vs SSC-H). Single cells were further gated depending on cell viability. Cells expressing B220 and IgM (B cells) or CD3 (T cells) were identified within the live cells gate (DCM-). CD8+ T cells and CD4+ T cells were identified within the CD3+ T cell gate.

☒ Tick this box to confirm that a figure exemplifying the gating strategy is provided in the Supplementary Information.
